# Dissecting molecular, pathological, and clinical features associated with tumor neural/neuroendocrine heterogeneity

**DOI:** 10.1016/j.isci.2023.106983

**Published:** 2023-05-28

**Authors:** Ling Cai, Ralph J. DeBerardinis, Guanghua Xiao, John D. Minna, Yang Xie

**Affiliations:** 1 Quantitative Biomedical Research Center, Peter O’Donnell School of Public Health, UT Southwestern Medical Center, Dallas, TX 75390, USA; 2 Children’s Research Institute, UT Southwestern Medical Center, Dallas, TX 75390, USA; 3 Simmons Comprehensive Cancer Center, UT Southwestern Medical Center, Dallas, TX 75390, USA; 4 Howard Hughes Medical Institute, University of Texas Southwestern Medical Center, Dallas, TX 75390, USA; 5 Department of Bioinformatics, University of Texas Southwestern Medical Center, Dallas, TX 75390, USA; 6 Hamon Center for Therapeutic Oncology Research, University of Texas Southwestern Medical Center, Dallas, TX 75390, USA; 7 Department of Pharmacology, UT Southwestern Medical Center, Dallas, TX 75390, USA; 8 Department of Internal Medicine, University of Texas Southwestern Medical Center, Dallas, TX 75390, USA

**Keywords:** Computational bioinformatics, Cancer systems biology, Cancer

## Abstract

Lineage plasticity, especially transdifferentiation between neural/neuroendocrine (NE) and non-NE lineage, has been observed in multiple cancer types and linked to increased tumor aggressiveness. However, existing NE/non-NE subtype classifications in various cancer types were established through ad hoc approaches in different studies, making it difficult to align findings across cancer types and extend investigations to new datasets. To address this issue, we developed a generalized strategy to generate quantitative NE scores and a web application to facilitate its implementation. We applied this method to nine datasets covering seven cancer types, including two neural cancers, two neuroendocrine cancers, and three non-NE cancers. Our analysis revealed significant NE inter-tumoral heterogeneity and identified strong associations between NE scores and molecular, histological, and clinical features, including prognosis in different cancer types. These results support the translational utility of NE scores. Overall, our work demonstrated a broadly applicable strategy for determining the NE properties of tumors.

## Highlights

• We developed a webtool that employs a generalized strategy to generate NE scores
• We evaluated NE heterogeneity in seven cancer types
• We examined NE association with molecular, histological, and clinical features of cancer
• NE score associates with outcome in glioma and medulloblastoma

## Graphical abstract

**Figure:**
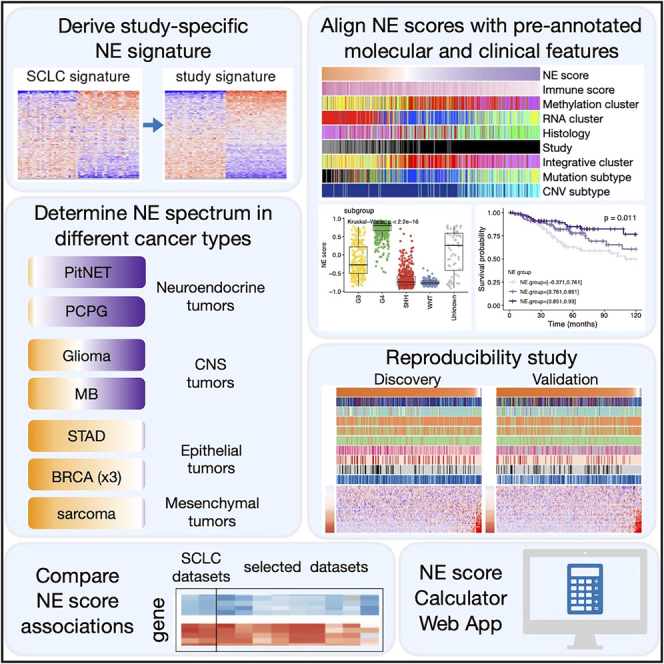

## Introduction

Lineage plasticity is a relatively new hallmark of cancer,^1^ whereby cancer cells can switch between different lineages. One notable example is the transdifferentiation from neural/neuroendocrine (NE) to non-NE states that has been observed in several types of NE cancer. For instance, in small-cell lung cancer (SCLC), a high-grade neuroendocrine cancer, two distinct states were initially observed based on the distinct morphology and growth properties in culture: “classic”/“neuroendocrine” and “variant”/“non-neuroendocrine.”^2^ Similarly, in high-grade glioma, two distinct states were identified as “proneural” and “mesenchymal,” which were found to have different prognoses in patients.^3^ In neuroblastoma (NBL), these two states were characterized as “adrenergic” and “mesenchymal.”^4^ Conversely, the transdifferentiation from non-NE to NE states has been established as a mechanism of therapeutic resistance in a few non-NE cancer types. For example, in lung adenocarcinoma, this confers resistance to EGFR inhibitors; while in prostate cancer, this confers resistance to hormonal therapy or castration.^5^

However, as the NE/non-NE subtype classifications were established by ad hoc approaches in different cancer types by different studies, it is difficult to align these findings, and the appreciation of lineage plasticity is often siloed within specific cancer types. By projecting pan-cancer epithelial tumors to a principal component analysis framework established by lung and prostate non-NE and NE tumors, a previous study has identified subsets of NE tumors in non-NE epithelial cancers, but the performance of this approach on expression data generated from different platforms has not been assessed and no clear reference range for NE and non-NE tumors has been established.^6^

We previously established an NE score calculation method for SCLC samples based on a gene expression signature derived from differentially expressed genes in SCLC and non-NE lung cancer cell lines.^7^^,^^8^ From a comparative study of NE heterogeneity in SCLC and NBL, we found the SCLC NE signature works surprisingly well in NBL, and the molecular features associated with NE transdifferentiation are highly conserved in these two distinct types of cancer,^9^^,^^10^ inspiring us to extend this method to study NE features of other cancer types.

While applying the SCLC-derived NE signature to other cancer types for NE scoring may be convenient, it is important to consider the tissue/organ context because tumors are abnormal growths that can hijack gene expression modules specific to the tissue/organ from which they originated. This means that the genes expressed in a tumor are tools used by the biological processes driving carcinogenesis, rather than independent actors. For example, neuroblastoma, pheochromocytoma, and paraganglioma are originated from the neural crest and their NE programs may reflect certain embryonic cell populations in the neural crest; in SCLC, prostate cancer, and gastrointestinal neuroendocrine tumors, the cancer cells may share NE program similar to that of the tissue-specific normal NE cells that carry out sensory and neuropeptide-secreting functions. Therefore, a cancer type-specific NE score should reflect the specific transcriptional program for NE and non-NE lineage within the tissue context.

To account for this tissue specificity, we developed a generalized strategy to calculate cancer type-specific NE scores and applied it to seven cancer types. We investigated the association between NE scores vs. existing molecular classifications and clinical features. An outline of this study is provided in Figure S1.

## Results

### Deriving cancer-specific NE signatures that preserve the lineage-differentiating abilities

First, we set out to identify cancer types with some degree of inter-tumoral NE heterogeneity from published datasets. We applied the original SCLC NE signature to characterize inter-tumoral NE heterogeneity in the pan-cancer samples from the Cancer Genome Atlas (TCGA) project^11^ and identified six cancer studies that each had more than five cases with positive NE scores (Figure S2A). Five of these were selected for follow-up analysis. The pancreatic adenocarcinoma (PAAD) dataset was excluded because the identified NE tumors were not included in the original PAAD study^12^ (Figure S2B). To provide examples of independent cohort validation, we included the Metabric discovery and validation datasets^13^ to confirm and extend the findings from the TCGA breast cancer (BRCA) dataset. In addition to TCGA studies, we included datasets from medulloblastoma (MB)^14^ (neuroectodermal cancer), pituitary neuroendocrine tumor (PitNET),^15^ and high-grade soft tissue sarcomas^16^ (non-NE cancer), such that we have a balanced selection of three non-NE, two neural, and two neuroendocrine cancer types in our panel.

**Figure 1:**
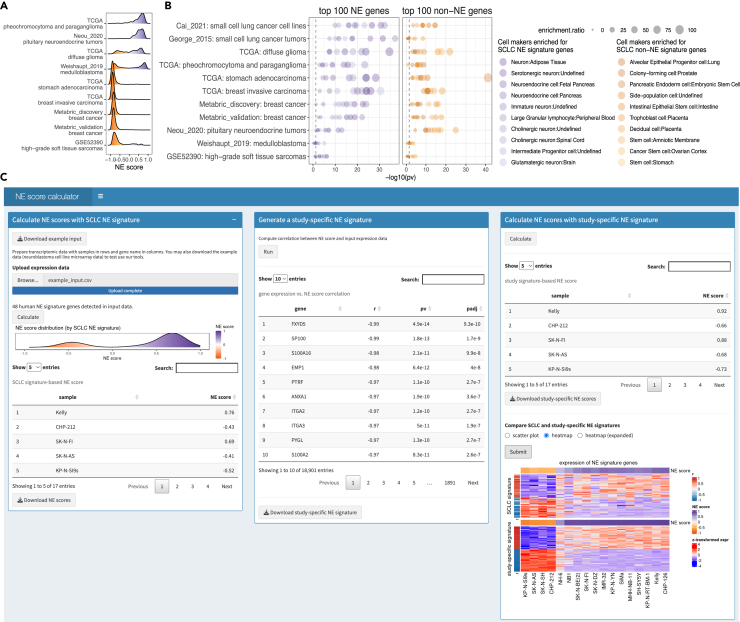
Deriving cancer-specific NE signatures from an SCLC NE signature (A) Distribution of NE scores in nine selected datasets from seven cancer types. These NE scores were computed with cancer-specific NE signatures. (B) Pathway analyses for top cancer-specific NE and non-NE genes. Dash lines indicate p values of 0.05. (C) NE score calculator at https://lccl.shinyapps.io/NEcalc/.

Although most of the NE and non-NE genes derived from the SCLC cell line datasets were also differentially expressed in these selected datasets (Figure S3A, left side of each heatmap), to obtain more robust study-specific gene signatures, we performed correlations between NE scores derived from the SCLC signature and the transcriptomic data from each study. The top 25 positively correlated (NE) genes and the top 25 negatively correlated (non-NE) genes were included to make a new signature (Data S1). We confirmed the new signature genes were distinctively expressed in NE vs. non-NE tumors (Figure S3A, right side of each heatmap). The majority of the NE and non-NE genes from the original SCLC signature are still highly ranked in the study-specific NE score-correlated genes (Figure S3B). These study-specific NE signatures were then used to generate new NE scores for follow-up analyses (Figure 1A). The new NE scores are highly correlated with the old NE scores but have broader ranges (Figures S3C and S3D). To compare the biological relevance of these study-specific signatures with the original SCLC signature, we performed pathway analyses using the cell type signature gene set library. We first identified pathways enriched for NE and non-NE genes, respectively, in the original SCLC signature. As expected, the top 10 cell marker gene sets associated with the NE genes are related to NE cell lineage, whereas the top 10 gene sets associated with the non-NE genes have a strong “stem cell” flavor. We then assessed the enrichment of these selected cell marker gene sets in study-specific NE and non-NE genes. Statistically significant results were obtained from all datasets, and the results are comparable to those from the SCLC cell line and tumor datasets (Figure 1B), suggesting that these newly derived cancer-specific NE signatures have retained the lineage-differentiating abilities of the original NE signature.

We developed a web application to facilitate the calculation of NE scores using our generalized strategy (Figure 1C). The tool is compatible with both human and mouse transcriptomic datasets. Users may upload their own dataset or use our example data for testing. The calculations are implemented in a stepwise manner and the tool alerts users if their input dataset lacks sufficient NE heterogeneity to generate reliable results (Figure S4). In step 1, NE scores based on the SCLC NE signature are computed and can be downloaded. In step 2, the correlation between the SCLC NE score and all genes in the transcriptome is calculated. The top 25 correlated and 25 anti-correlated genes are selected to make a study-specific NE signature that can be downloaded. In step 3, new NE scores computed from the study-specific NE signature are calculated and can be downloaded. Users may also compare the old and new NE scores in a scatterplot or a heatmap (Figure 1C).

### NE scores are associated with distinctive molecular, clinical, and pathological features in different cancer types

As previous TCGA studies have already comprehensively curated clinical and pathological features of the tumors and established molecular classifications for different cancer types based on large-scale multi-omics profiling data, we tried to dissect the relationship between these more well-known features and the previously overlooked NE features. Based on our prior knowledge about the transdifferentiation process between NE and non-NE cancer subtypes in SCLC, we selected genes pathways of interest and evaluated their correlation with study-specific NE scores to assess inter-cancer type consistency. In SCLC, the non-NE subtype exhibits Notch and Hippo activation.^7^ Neuronal genes that are targets of transcriptional repressor restrictive element-1 silencing transcription factor (REST) are highly expressed in the NE subtype of SCLC, whereas interferon-stimulated genes (ISGs) are highly expressed in the non-NE subtype.^8^ We defined consensus gene sets for these pathways (Figure S5). We included the Myc oncogene family, as Myc was found to be frequently activated in the variant SCLC subtypes. We also derived MHC I scores and examined the genes from an interferon-gamma signature that predicts response to immunotherapy^17^ to gauge the “immune hotness” of the tumors. In the following sections, we present seven case studies of NE score association with molecular, clinical, and pathological features in cancer-specific contexts. Results from these associations are also detailed in Data S2.

#### Diffuse glioma (CNS cancer)

**Figure 2:**
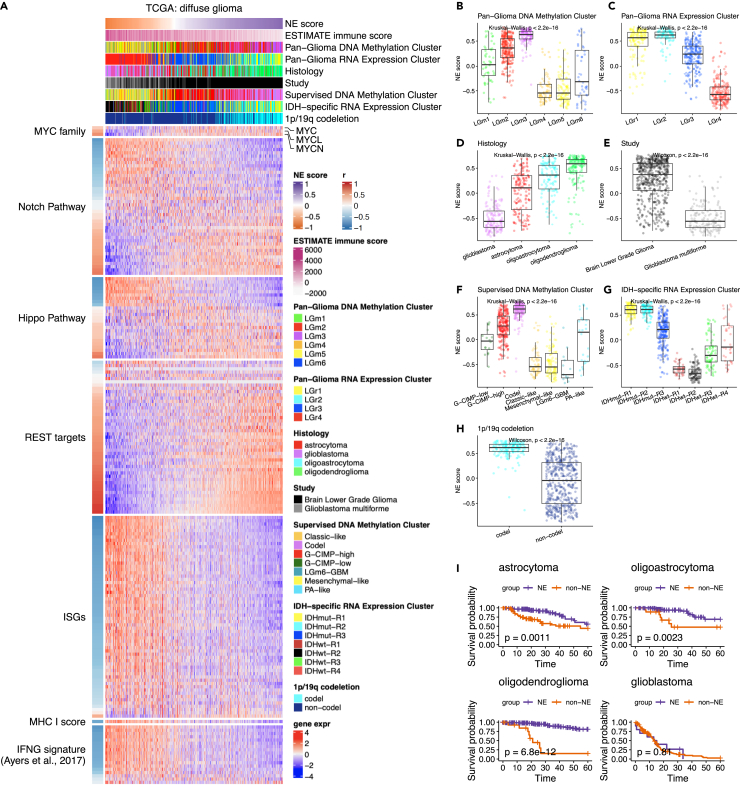
Aligning NE scores to various features of diffuse glioma (A) Heatmap aligning NE scores to selected features in diffuse glioma. (B–H) Comparison of NE scores across different methylation clusters (B), RNA clusters (C), histology subtypes (D), studies (E), supervised clusters (F), IDH clusters (G), and 1p/19q co-deletion status (H). (I) Lower NE scores associated with worse overall survival in low-grade gliomas.

In TCGA’s publication, brain low-grade glioma (LGG) and glioblastoma multiforme (GBM) were analyzed collectively as diffuse glioma.^18^ We derived NE scores for 662 diffuse glioma tumors. These NE scores vary significantly across subclasses defined by RNA expression and DNA methylation data (Figures 2A–2C), as well as by histological subtype, IDH mutation status, 1p/19q co-deletion status, estimated immune infiltrates, etc. (Figures 2A, 2D–2H, S6A, and S6B). The NE scores were much lower in GBM than in LGG, and lower in IDH wt compared to IDH mut. Notably, these NE scores are strongly prognostic, with lower NE scores associated with worse survival. Although NE scores are generally higher in the low-grade gliomas compared to glioblastoma (Figure 2E), low NE scores within each low-grade glioma subtype are still associated with worse survival (Figure 2I). The IDH-mutant tumors are known to be more indolent than IDH-WT tumors of the same grade, yet low NE scores are still significantly associated with poor survival in IDH-WT tumors (p value = 0.014) and close to significantly associated with poor survival in IDH-mutant tumors (p value = 0.079) (Figure S6C). Additionally, across all the clinical and molecular features published in the original study, we identified 31 numerical and categorical prognostic features using univariate Cox proportional-hazards (PH) regression. When the NE score was added as a covariate in a multivariate Cox PH model with each of these prognostic features, the outcome associations for NE score remained significant in all but one model (Figure S7). This only exception was the supervised cluster feature that integrated inputs from IDH mutation status, histology, DNA methylation cluster, and DNA methylation levels (Figures S7A and S7E). These results suggest NE score is a strong prognostic factor in glioma and that non-NE features in low-grade gliomas may mediate tumor aggressiveness.

#### Medulloblastoma (MB, CNS cancer)

**Figure 3:**
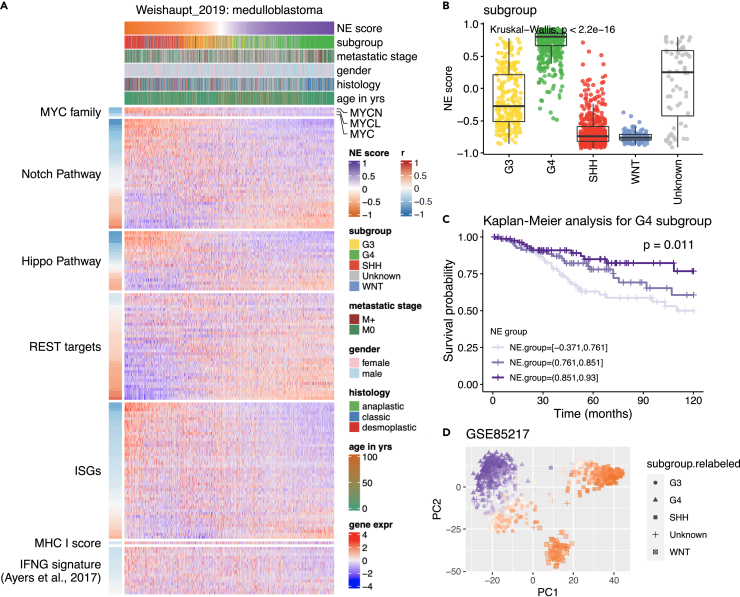
Aligning NE scores to various features of medulloblastoma (MB) (A) Heatmap aligning NE scores to selected features in MB. (B) Comparison of NE scores across different molecular subtypes. (C) Kaplan-Meier analysis of NE score association with overall survival in G4 subgroup of MB. Patients were stratified into low, medium, and high NE score groups with equal sizes. (D) Relationship between NE score and MB tumor expression profile as assessed by principal component analysis. G3 and G4 tumors exist along a transcriptional continuum as previously reported. Low-NE-score G4 tumors are more similar to G3 tumors.

In addition to diffuse gliomas, we examined MB, another type of central nervous system tumor that often grows at the cerebellum. We used the batch normalized data that contain 1,641 tumors from 25 studies.^14^ The distribution of NE scores in this combined MB dataset is similar to the diffuse glioma cohort, with a little more than half of the samples having positive NE scores (Figure 3A). NE scores also differed by MB subtypes (Figure 3B). G4 tumors had more positive NE scores whereas WNT and SHH tumors had more negative NE scores. G3 subtype of MB is known to be the most aggressive. Interestingly, the NE scores of G3 tumors were the most broadly distributed. This pattern was consistently observed across the individual studies (Figure S8A). Given the high prognostic value of NE scores in diffuse gliomas, we performed survival analyses to assess the prognostic value of NE scores in MB. The MB subtype was strongly associated with prognosis, but the association between NE scores and survival was statistically insignificant (Figure S8B). To look for subtype-specific survival association, we ran a CoxPH multivariate model including NE score and subtype as predictor variables with interaction. A statistically significant interaction was found between NE score and G4 subtype (Figure S8C). We visualized this finding using a Kaplan-Meier plot generated from only patients with G4 tumor (Figure 3C). Examining the NE score–survival association in patients with G4 tumor while controlling for metastatic stage, sex, and age, we found the association remained statistically significant, with lower NE scores associated with worse survival (Figure 3D). As G3 and G4 tumors are known to exist along a transcriptomic continuum,^19^ we examined the relationship between NE score and the expression similarity in GSE85217 MB samples and found the low-NE-score G4 samples are transcriptionally more similar to the G3 tumors (Figure S8D).

#### Pituitary neuroendocrine tumor (PitNET, NE cancer)

**Figure 4:**
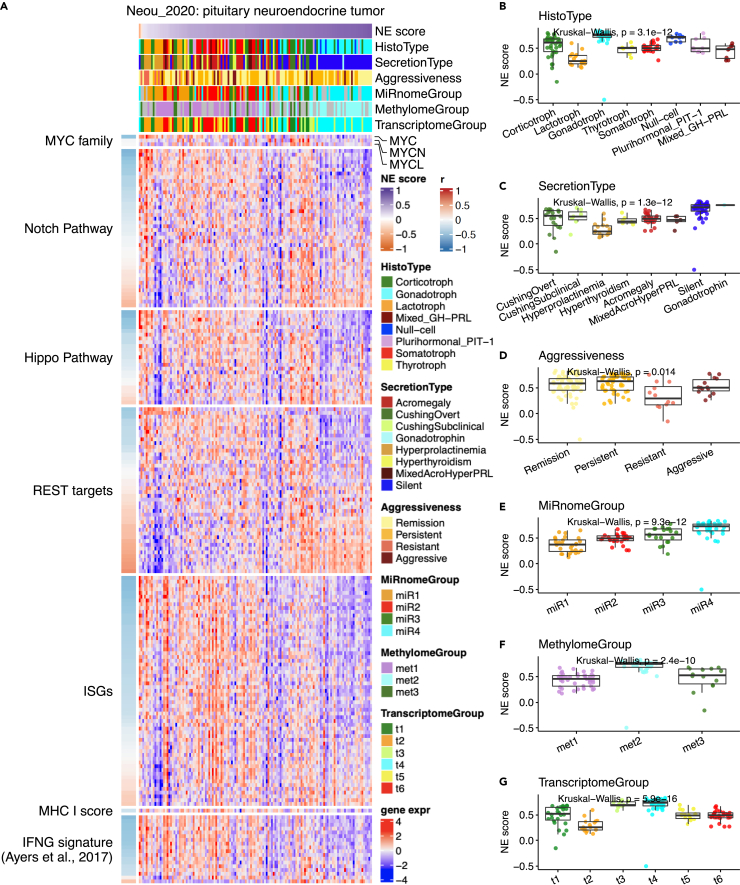
Aligning NE scores to various features of pituitary neuroendocrine tumor (PitNET) (A) Heatmap aligning NE scores to selected features in PitNET. (B–G) Detailed comparison of NE scores across different histological subtypes (B), secretion types (C), disease aggressiveness (D), subgroups defined by miRNA (E), methylome (F), and transcriptome (G).

We generated NE scores for 134 PitNETs^15^ (Figure 4). Only two samples with negative NE scores were identified in this PitNET cohort but the PitNET NE scores were still strongly associated with histological and molecular subtypes (Figures 4B–4G). Tumors with the highest NE scores belonged to the gonadotroph histological subtype and the silent secretion type. These tumors are also less aggressive. Tumors with relatively low NE scores belong to the lactotroph histological subtype and hyperprolactinemia secretion subtype. These tumors were more resistant. However, we did not observe any distinctive patterns in the negative-NE-score tumors.

#### Pheochromocytoma and paraganglioma (PCPG, NE cancer)

PCPG are also neuroendocrine cancers. We generated NE scores for 171 PCPG tumors. While most samples had high NE scores, we observed robust associations between NE scores and subtypes defined by mRNA, DNA methylation, miRNA, copy number, and RPPA data (Figure S9). NE scores also differed by several copy number alteration events (Figure S10A). However, NE scores do not differ by tumor type (PC vs. PG) or tumor aggressiveness. Samples with low NE scores were also found to have low purity (Figure S10B). Unlike the pattern we previously observed for SCLC^8^ or diffuse glioma in this study (Figure 2), in PCPG, subsets of samples with high NE scores seemed to have consistently high expression of ISGs and IFNG signature genes (Figure S9A). These samples were identified as outliers in a linear model regressing the average scaled expression of the selected ISGs on the NE scores (Figure S11A). Interestingly, the residuals from this model were highly correlated with the “Leukocyte Index Methylation” feature from the original study (Figures S11B and S11C), suggesting that the expression of these ISGs that cannot be explained by the tumor NE score could be attributed to the infiltrating leukocytes.

#### Breast cancer (BRCA, non-NE cancer)

**Figure 5:**
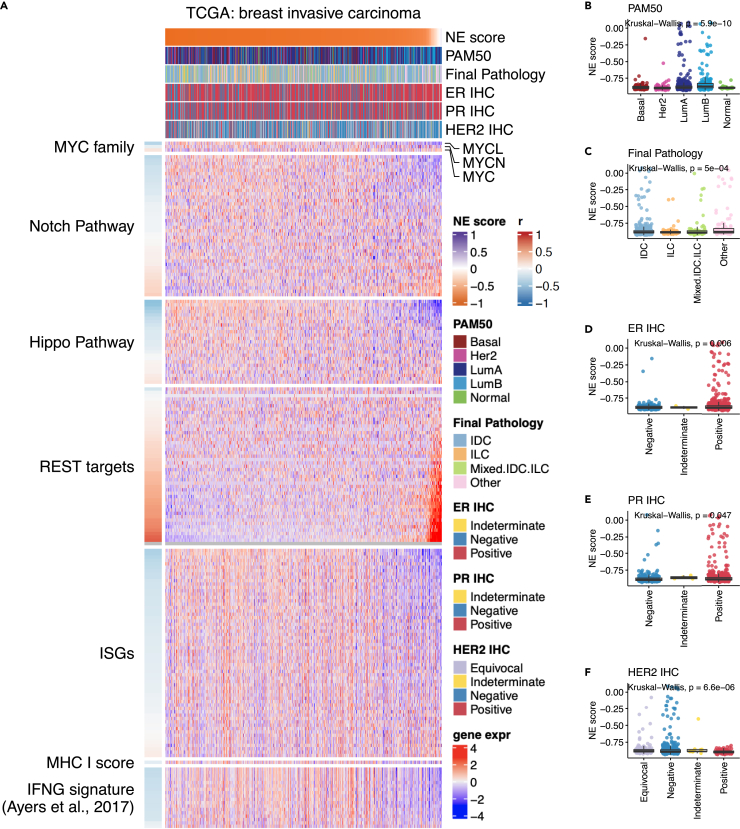
Aligning NE scores to various features of breast invasive carcinoma (BRCA) (A) Heatmap aligning NE scores to selected features in BRCA. (B–F) Comparison of NE scores across different PAM50 clusters (B), pathological clusters (C), IHC status of ER (D), PR (E), and HER2 (F).

We investigated 814 BRCA tumors that can be matched to samples included in the original study^20^ (Figure 5A). Of these tumors, seven (<1%) have positive NE scores. But strong associations were observed between NE scores and molecular subtypes (Figures 5B–5F). The high-NE-score samples were enriched in lumA and lumB of the PAM50 subtypes and in ER + HR-tumors, consistent with existing knowledge about neuroendocrine breast cancers (NEBC).^21^ We also analyzed datasets from Metabric, another large-scale omics profiling study on breast cancer (Figures S12). The Metabric data were released in two cohorts, discovery and validation, each with close to one thousand samples. We used the Metabric discovery dataset to derive an NE gene signature and applied this signature to calculate NE scores for both the discovery and validation datasets. The heatmaps generated from these two cohorts were highly similar (Figure S12A) and similar to the heatmap from the TCGA BRCA cohort (Figure 5A). In Metabric, there were 18 and 28 positive NE-score samples in the discovery and validation cohort, respectively (2%–3%). In addition to the confirmation that NEBC tumors are enriched in the ER + HR-luminal invasive ductal subtype, our analyses of the Metabric datasets also revealed more frequent high-NE-score tumors in postmenopausal women (Figures S12B and S12C), which is also consistent with previous literature.^22^

#### Stomach adenocarcinoma (STAD, non-NE cancer)

We also investigated 277 STAD tumors that could be matched to samples included in the original study^23^ (Figure S13A). The number of high-NE-score samples was very small; only three samples had positive NE scores, all belonging to the tubular class (Figure S13B). However, we were still able to identify robust associations between NE scores and molecular subtypes (Figures S13C–S13G). The high-NE-score samples belong to the chromosome instability molecular subtype (Figure S13C) as they had high levels of copy number alterations (Figure S13H). However, they were not microsatellite unstable, hypermutated, EBV positive, or epigenetically silenced in *CDKN2A* or *MLH1*, and did not have mutations on *PIK3CA*, *RHOA*, *ARID1A*, or *KRAS* (Figures S13I–S13Q).

#### High-grade soft tissue sarcoma (non-NE cancer)

As the last non-NE cancer we studied, we investigated 79 sarcoma samples belonging to 8 sarcoma subtypes (Figure S14A).^16^ As sarcomas arise from mesenchymal cells of origin, most samples had negative NE scores. However, many synovial sarcoma (SS) tumors were found to have high NE scores. This is interesting because SS mimicry of neuroendocrine tumors has been noted in the literature before.^24^ Malignant peripheral nerve sheath tumors (MPNST) also exhibit highly variable NE scores, with a couple of positive-NE-score samples, perhaps reflecting their neural origin (Figure S14B). As both SS and MPNST are commonly found in children and adolescents, we also observed a negative correlation between NE scores and age (Figure S14A).

### NE score correlations are conserved across different cancer types

**Figure 6:**
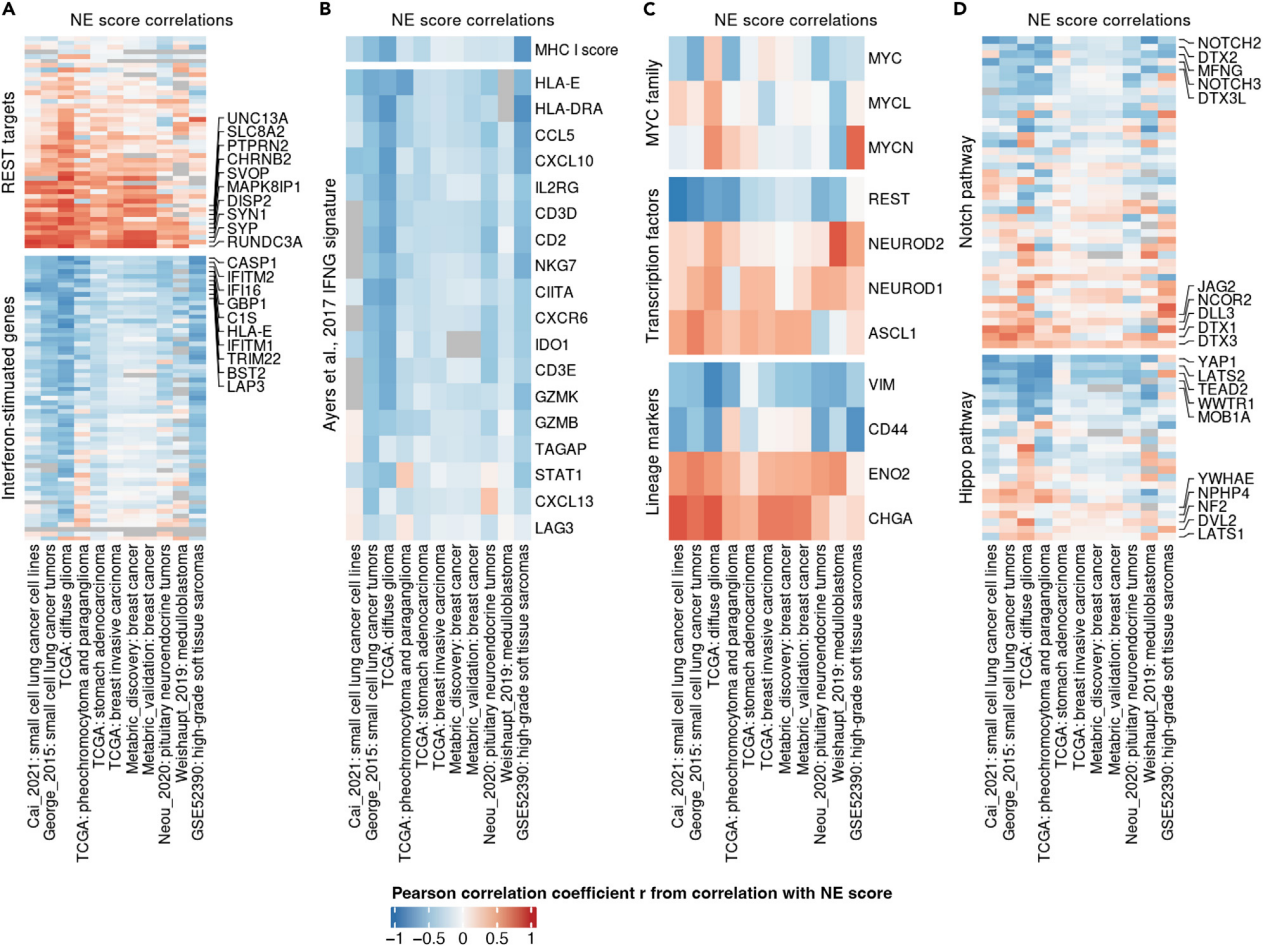
Cross-study comparison of NE score correlations Heatmaps comparing the correlation between NE scores and expression of genes from REST targets and ISGs (A), members of an IFNG signature that predicts response to immunotherapy (B), MYC family members, NE lineage-related transcription factors, NE and non-NE lineage markers (C), and consensus members of Notch and Hippo pathways (D).

Having generated NE scores for 5,772 tumor samples from nine datasets covering seven cancer types, we next compared the cross-cancer type consistency of NE score-associated gene expression (Figure 6). The results from an SCLC cell line dataset and an SCLC tumor dataset were also added to the heatmaps for reference. We found consistent positive correlations with at least half of the consensus neuronal genes targeted by the transcriptional repressor REST and consistent negative correlations with ISGs (Figure 6A), in agreement with our previous observations in SCLC.^8^ Also consistent with our previous finding that high-NE-score tumors are immune cold tumors,^8^ anti-correlations were found between NE score and MHC I scores, and between NE scores and IFNG response signature genes across studies (Figure 6B). Although *MYC* amplification was found to be frequent in the variant subtype of SCLC, we did not observe a consistent pattern between NE scores and Myc family genes (Figure 6C). In the examination of lineage-specific transcription factors and markers, we found consistent negative correlations for the repressor transcription factor *REST* and consistent positive correlations for the neuro/neuroendocrine lineage drivers *ASCL1*, *NEUROD1*, and *NEUROD2*. Expression of the mesenchymal marker vimentin and stem cell marker *CD44* negatively correlated with NE scores in over half of the datasets. In contrast, neuron-specific enolase and chromogranin A, two clinically used markers for neural/neuroendocrine tumors consistently exhibit positive correlations with NE scores across studies (Figure 6C). Mixed results were obtained when we examined the correlations for consensus Notch and Hippo pathway genes across studies (Figure 6D). Among the more consistent results, Notch receptors (*NOTCH2* and *NOTCH3*) negatively correlated with NE scores, whereas Notch inhibitors (*DLL3*, *DTX1*, and *DTX3*) positively correlate with NE scores, consistent with previous findings that Notch activation mediates NE to non-NE transdifferentiation in SCLC.^25^ *YAP1*, the Hippo pathway transcription factor that defines one subtype of non-NE SCLC tumors,^26^ exhibited the most consistent negative correlation with NE scores across studies. While we only included a selected panel of genes in this analysis, we provide NE score–gene expression correlation results for the full transcriptome in Data S3. Overall, our results suggest that differentially expressed genes between NE and non-NE tumors are moderately conserved across different cancer types.

## Discussion

In this study, we developed a generalized strategy to determine NE features in cancer samples based on the premise that NE genes (neural, neuroendocrine) and non-NE genes (stem cell, mesenchymal) assume opposite transcriptional regulation and lineage transition along the NE/non-NE axis shifts the NE and non-NE programs in opposite directions. This premise is based on the previous findings in SCLC that YAP and REST corroborate to mediate NE lineage plasticity.^27^ In non-NE cancer cells, REST and YAP are highly expressed to repress NE fate. While REST/NRSF (neuron-restrictive silencing factor) is a master repressor of the NE differentiation.^28^ As effectors of the Hippo signaling pathway, YAP also plays roles in controlling stem/progenitor cell differentiation and hence upregulates such programs in the non-NE cells.^29^ Considering the NE and non-NE genes in different tissue types or cancer types may be somewhat different; taking the SCLC-specific NE signature as a lead, we identified study-specific NE signatures in seven cancer types and verified that these new signatures have preserved the lineage-differentiating properties (Figures 1 and S3). While our approach worked well for these seven cancer types, for researchers to adopt this strategy to study other cancer types, one should verify that the samples in the new dataset exhibit adequate variations in NE scores (as first determined by the SCLC NE signature) to call NE score-correlated genes and the majority of the original NE and non-NE genes from the SCLC signature are differentially expressed in the new dataset (Figure S4B).

Taking this generalized NE scoring approach, we identified subsets of NE tumors in typically non-NE cancers (BRCA, STAD, and high-trade soft tissue sarcomas) and subsets of non-NE tumors in typically NE cancers (PiNET and PCPG), while the original studies had no mention of subtypes along the NE/non-NE spectrum. We showed that, in each cancer type, NE and non-NE outliers exist and NE scores are associated with distinctive molecular subtypes and clinical features. We have demonstrated that such associations are highly reproducible in some cancer types, such as the validation of TCGA BRCA results using the Metabric datasets (Figures 5 and S12), and the consistent NE score distribution pattern across MB subtypes in multiple MB datasets (Figure S8A). However, we did not perform validation for every cancer type studied in this paper, and this paper only covers a limited number of cancer types. It would be important to further validate our findings in other cancer types in future studies. In particular, it would be interesting to apply this approach to study tumors from the gastroenteropancreatic organs where neuroendocrine neoplasms are often found.

In our analyses, we found that lower NE scores were associated with worse outcomes in two neural cancers: low-grade glioma (Figure 2I) and the G4 subtype of MB (Figure 3C). Notably, in the analysis of TCGA diffuse glioma samples, although low-grade glioma generally had lower NE scores than the more aggressive GBM, lower NE scores were still strongly associated with worse prognosis within low-grade glioma subtypes while controlling for known prognostic factors such as IDH mutation status and 1p/19q co-deletion status. While it is interesting that tumors with lower NE scores are more aggressive, it remains to be dissected whether this was due to a deprogramming of neural gene expression or an increased composition of non-neural cells in the milieu.

In MB, we found the most aggressive G3 tumors also exhibited the most variable NE scores, suggesting their higher lineage plasticity than the other subgroups. A previous study has suggested that the G3 and G4 MB tumors exist as intermediates on a bipolar continuum between archetypal group 3 and group 4 entities.^19^ In this study, we found that low-NE-score G4 tumors experience worse outcome (Figures 3C and S8D), and these G4 tumors are also more similar to the G3 tumors. It is therefore possible the low-NE-score G4 tumors also exhibit higher lineage plasticity that promotes disease progression. In SCLC, it has been shown that Myc mediates lineage transition from NE to non-NE fate.^30^^,^^31^ Interestingly, Myc amplification is also known to be enriched in G3 MB tumors.^32^ It would be interesting to determine whether Myc also plays a role in the NE plasticity in the G3 tumors. Taken together, our findings suggest NE score may be clinically useful in predicting patient outcomes in low-grade glioma and MB. However, it would be important to reduce the NE signature to a smaller, clinically achievable gene set for NE scoring to be incorporated into the existing risk evaluation scheme.

Notably, besides glioma and MB, we did not find significant associations between NE scores in other tumor types with outcome data available. This contrasts with a previous pan-cancer study that reported an association between high NE features and poorer prognoses in epithelial cancers.^6^ In SCLC, therapy resistance from lineage plasticity is attributed to the differential drug sensitivity possessed by co-existing cancer subtypes within the same tumor, rendering the tumor more resilient to standard therapy.^33^ However, the therapeutic implications of NE transdifferentiation may be highly context dependent, as treatment regimens vary across different cancer types, and the lineage reprogramming may confer additional vulnerability to cancer cells. It would be interesting to see future studies that perform prognosis associations in larger datasets with high NE heterogeneity for more cancer subtypes.

Overall, our showcase studies that aligned NE scores to existing molecular, pathological, and clinical features in seven cancer types demonstrated the feasibility of a broadly applicable strategy to determine the NE properties of tumors and study inter-tumoral NE heterogeneity from bulk tumor transcriptomic data. Our easy-to-use web tool will enable researchers to employ this method for their own studies.

### Limitations of the study

One important consideration of this study is that the approach has not been extensively validated across multiple cancer types. Additionally, the assumption of a binary classification of cancer cells as NE or non-NE may not hold true for certain cancer types, such as leukemia cells. Researchers should perform a preliminary assessment using the SCLC-based NE signature to evaluate NE score distribution in their sample set of interest. Samples with limited NE heterogeneity may not be suitable for cancer-specific NE signature generation using this approach. Further validation and careful consideration of cancer-specific characteristics are necessary to ensure the applicability of the approach in different contexts.

## STAR★Methods

### Key resources table

**Table:** 

REAGENT or RESOURCE	SOURCE	IDENTIFIER
**Deposited data**		

Pan-cancer TCGA RNA-seq data	Vivian et al.^11^	link
METABRIC data	Curtis et al.^13^	EGAS00000000083
PitNET data	Neou et al.^15^	EGAD00001004996
Medulloblastoma data	Weishaupt et al.^14^	GSE124814
High-grade soft tissue sarcoma	Renner et al.^16^	GSE52390
SCLC datasets	Cai et al.^8^	https://doi.org/10.5061/dryad.zgmsbcc8z

**Software and algorithms**		

R version 4.0.2	R Foundation for Statistical Computing	http://www.r-project.org/
Bioconductor		www.bioconductor.org

### Resource availability

#### Lead contact

Further information and requests for data and code should be directed to and will be fulfilled by the lead contact, Ling Cai (Ling.Cai@UTSouthwestern.edu)

#### Materials availability

This study did not generate new unique reagents.

### Method details

#### Generation of NE scores and MHC I scores

The SCLC signature is based on SCLC cell line RNA-seq data as previously described.^7^^,^^8^ A quantitative NE score can be generated from this signature using the formula: NE score = (correl NE – correl non-NE)/2 where correl NE (or non-NE) is the Pearson correlation between expression of the 50 genes in the test sample and expression of these genes in the NE (or non-NE) cell line group. This score has a range of −1 to +1, where a positive score predicts for NE while a negative score predicts for non-NE cell types. The higher the score in absolute value, the better the prediction. In this study, the cancer-specific signatures were derived from study-specific transcriptomic data. The SCLC NE signature-derived NE scores were computed and used to correlate with transcriptomic data. The top 25 correlated and top 25 anti-correlated genes were combined as the new study-specific signature. Instead of using the mean expression from the NE and non-NE class samples, we assigned equal weights (1 for NE and −1 for non-NE) in the study-specific NE signatures (Data S1). MHC I geneset is a union set from “GO_MHC_CLASS_I_PROTEIN_COMPLEX” and “GO_MHC_CLASS_I_PEPTIDE_LOADING_COMPLEX” from Gene Ontology geneset library from MSigDB. R package GSVA^35^ was used to compute MHC I scores by single sample GSEA (ssGSEA) method.^36^^,^^37^

#### Identification of samples with ISG expression that cannot be explained by NE scores in PCPG

A linear regression model was fitted with NE score as the predictor variable and average expression of selected ISGs as the response variable. The residuals were classified into high and low groups using Gaussian mixture modeling.^38^ Samples in the high groups are considered outliers from this linear model, with large variations in ISG expression that cannot be explained by NE scores (Figure S7A).

#### Pathway analysis

Cell type-specific gene set libraries were downloaded from MSigDB.^37^ We selected the 10 most enriched cell marker genesets from hypergeometric tests of SCLC NE and non-NE signature genes. Hypergeometric tests were then performed to assess the enrichment of the top 100 NE score correlated/anti-correlated genes in different cancer types for these selected cell markers.

#### Deriving consensus genesets

Consensus Notch and Hippo pathway genesets were curated from related genesets in MSigDB (http://www.gsea-msigdb.org/gsea/msigdb).^37^ Consensus REST targeted neuronal genes and ISGs were selected from Enrichr curated^39^ transcriptional targets from the ENCODE project^40^ and the LINCS project.^41^

#### Statistical analyses

All statistical analyses were performed by R,^42^ with the following packages other than those mentioned above: “data.table”, “ComplexHeatmap”,^43^ “RColorBrewer”, “openxlsx”,”gdata”, “survminer”, “survival”, “Cairo”, “ggplot2”, “GGally”, “ggpubr”, “ggrepel”, “gridExtra”, and “patchwork”.

## Data Availability

• Pan-cancer RNA-seq data from TCGA processed by Expectation-Maximization (RSEM) algorithm was downloaded from Toil xena hub.^11^ We downloaded the following datasets from the European Genome-phenome Archive (EGA): Metabric data with accession number EGAS00000000083,^13^ PitNET data with accession number EGAD00001004996.^15^ We downloaded the following datasets from the Gene Expression Omnibus (GEO) using R package GEOquery^34^: GEO: GSE124814,^14^ and sarcoma dataset GEO: GSE52390.^16^ Phenotype data were downloaded from the original publications. • Our web application at https://lccl.shinyapps.io/NEcalc/ is a shiny app deployed at the shinyapps.io servers. It is implemented through the following R packages: ‘shiny’, ‘shinyjs’, ‘shinydashboard’, ‘shinycssloaders’, ‘data.table’, ‘DT’, ‘ComplexHeatmap’, ‘ggplot2’, ‘ggridges’, and ‘RColorBrewer’. The source code is available at https://github.com/cailing20/NEcalc. • Any additional information required to reanalyze the data reported in this paper is available from the lead contact upon request.

## Supplemental information

Document S1. Figures S1–S14

Data S1. Cancer-specific NE signatures, related to Figure 1

Data S2. Cancer-specific NE clinical correlations, related to Figures 2, 3, 4, and 5

Data S3. Cancer-specific NE transcriptome correlations, related to Figures 2, 3, 4, 5, and 6
