# Supplementary material for: Dissecting molecular, pathological, and clinical features associated with tumor neural/neuroendocrine heterogeneity
Source: iScience. 2023 May 28;26(6):106983. doi: 10.1016/j.isci.2023.106983 (PMC10291506; doi:10.1016/j.isci.2023.106983)
Supplement: Document S1. Figures S1–S14 [file mmc1.pdf]

iScience, Volume *26*

Supplemental information

Dissecting molecular, pathological, and clinical features associated with tumor neural/neuroendocrine heterogeneity

Ling Cai, Ralph J. DeBerardinis, Guanghua Xiao, John D. Minna, and Yang Xie

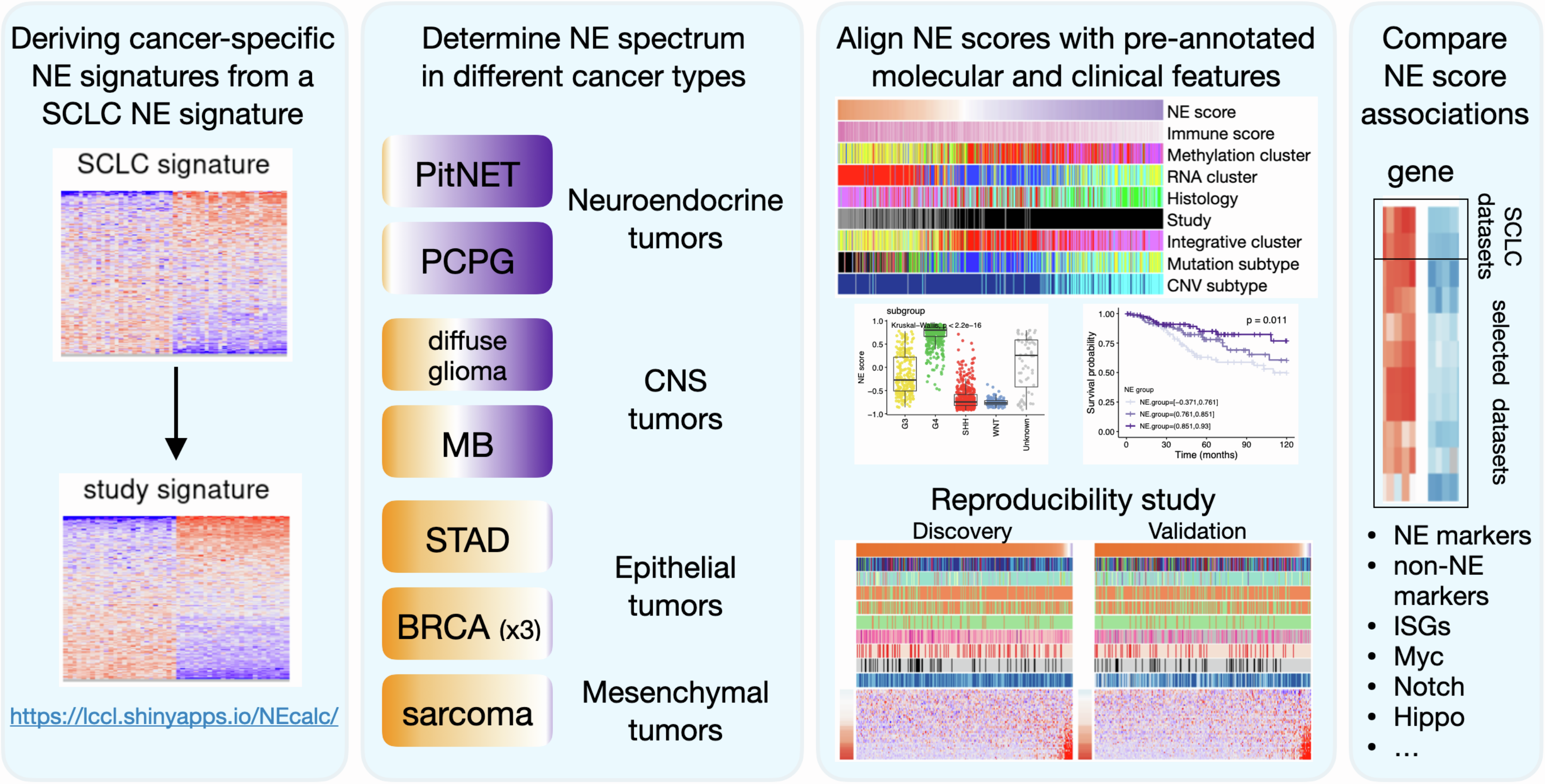

Figure S1. Study overview, related to Figure 1
We employed a generalized strategy to derive study-specific NE signatures based on a SCLC cell line-derived NE signature. We also developed a web application at https://lccl.shinyapps.io/NEcalc/ to help implement this method. We collected nine datasets for seven different cancer types and computed NE scores with cancer-specific NE signatures to determine the NE spectrum for each cancer type. For each cancer type, we aligned the existing molecular, clinical, and pathological annotations from published studies with NE scores to understand the relationship between NE and other cancer features. We validated the reproducibility of this approach using two breast cancer datasets. Finally, we compared the association between NE scores and selected genes of interest across different cancer types. Abbreviations: NE, neuroendocrine/neural; SCLC, small-cell lung cancer; PitNET, pituitary neuroendocrine tumor; PCPG, pheochromocytoma and paraganglioma; MB, medulloblastoma; STAD, stomach adenocarcinoma; BRCA, breast invasive carcinoma; CNS, central nervous system; ISG, interferon-stimulated gene.

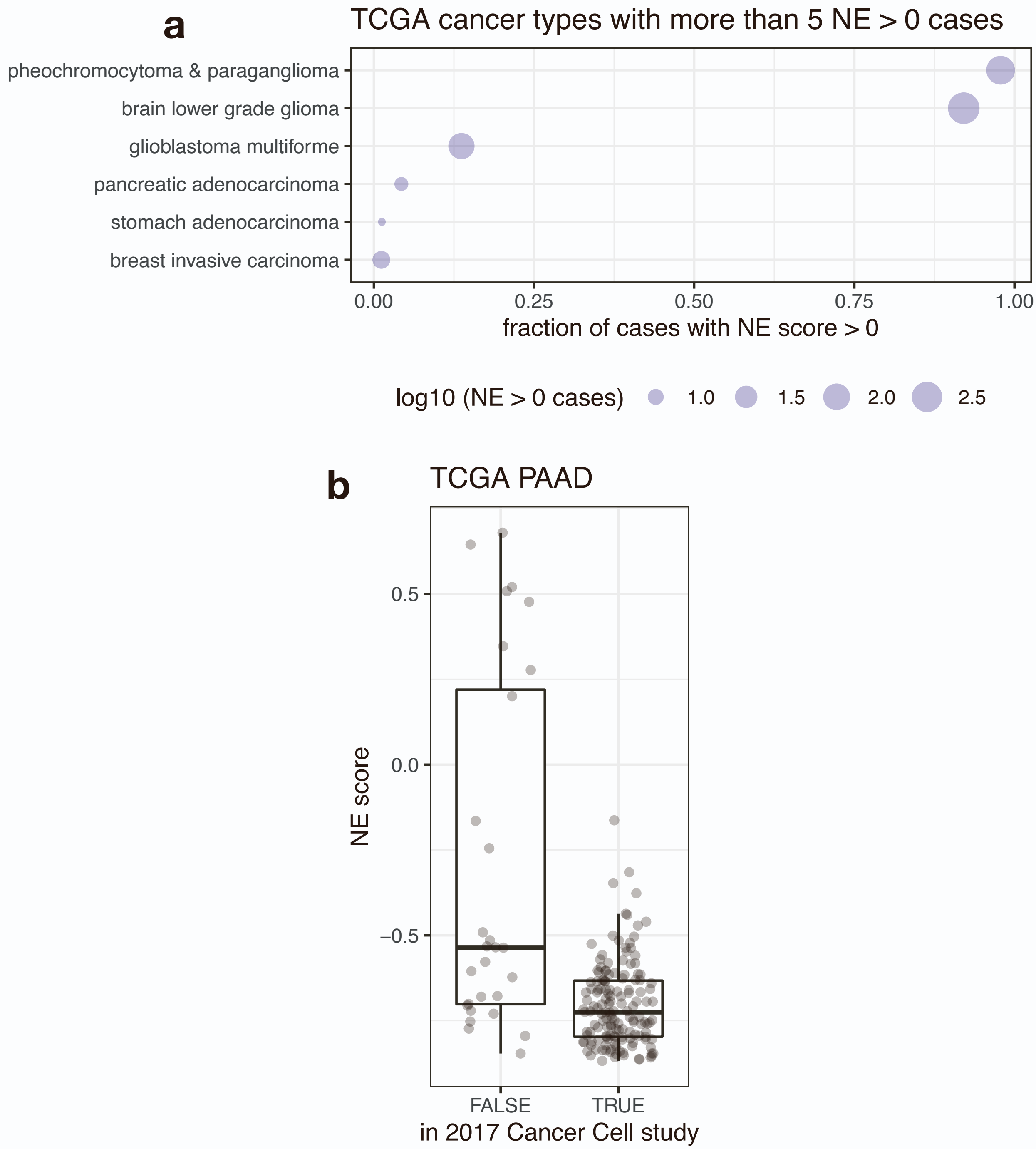

Figure S2. Selection of cancer types with mixed NE and non-NE tumors from TCGA studies, related to Figure1.

a, Six TCGA cancer types with at least five positive NE score samples were identified. These were selected from 12,804 TCGA tumor samples covering 33 cancer types. **b**, We excluded the TCGA PAAD dataset because the high-NE-score samples were not included in the original study. None of the eight PAAD tumors with positive NE scores were included in the 2017 study.

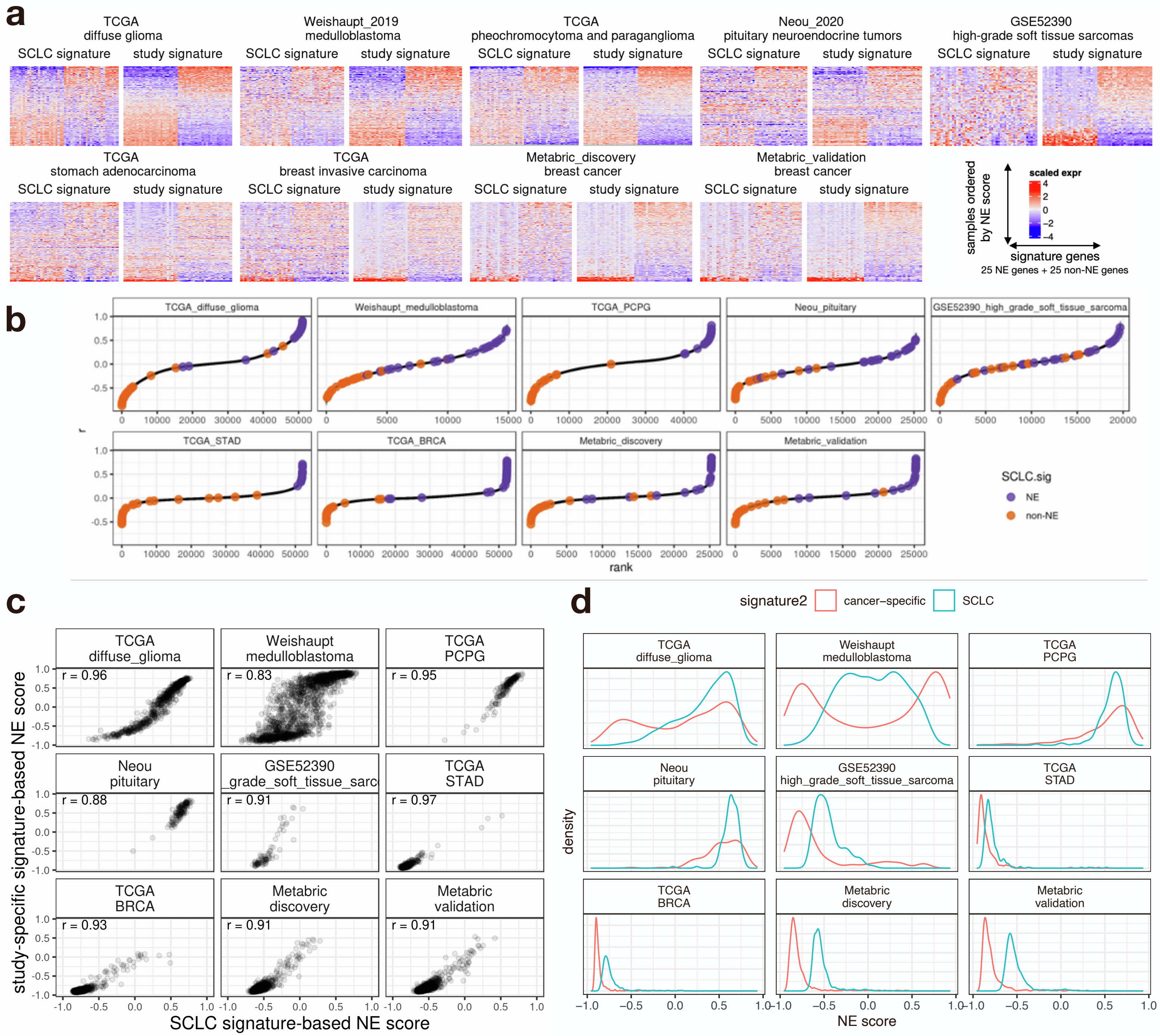

Figure S3. Comparison of NE scores calculated from SCLC NE signature and cancer-specific NE signature, related to Figure 1. **a**, Comparison of expression patterns from the original SCLC NE signature genes and cancer-specific NE signature genes. For each cancer type, a pair of heatmap for z-transformed expression data was plotted, with the left-side plot showing expression from the SCLC signature genes and the right-side plot showing expression from the cancer-specific signature genes. Samples in the rows of the heatmap were ordered by NE scores with low scores on top and high scores at the bottom. Columns of the heatmaps correspond to NE and non-NE genes from the 50-gene signature. **b**, Ranking of the 25 NE and 25 non-NE genes from the SCLC signature in study-specific NE score correlated genes. **c-d**, Comparison of NE scores generated from original SCLC signature and cancer-specific signature. In the scatter plot (**c**), the SCLC signature-based NE scores were plotted as x-axis values, the cancer-specific signature-based NE scores were plotted as y-axis values. Pearson correlation coefficient was calculated and provided in the upper left corner of the plot. In the density plot (**d**), the distribution of NE scores generated from SCLC (blue) and cancer-specific (red) NE signatures are compared.

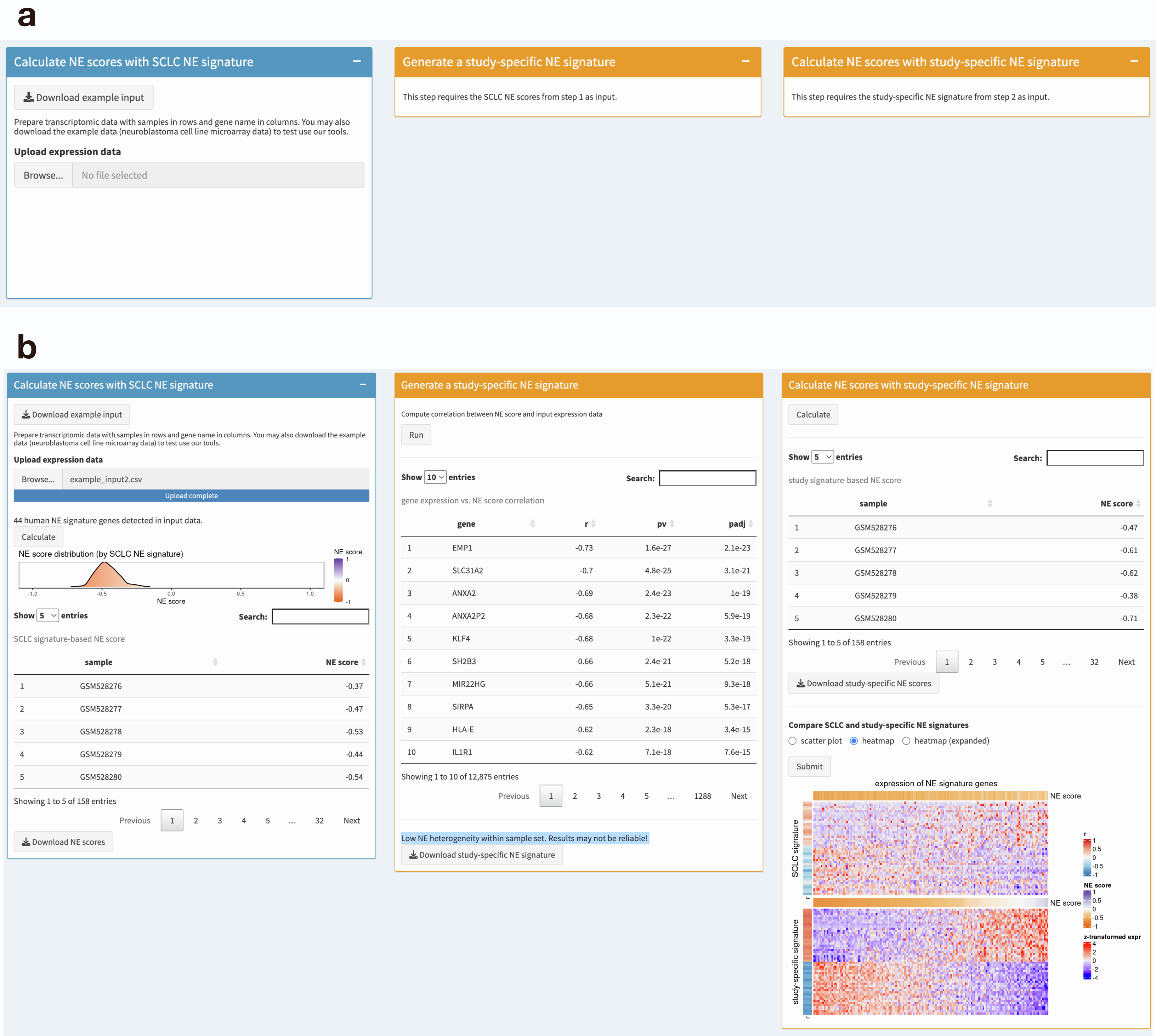

Figure S4. Step-wise determination of study-specific NE signature, related to Figure 1.
a, Users are required to upload their expression dataset in step 1 before they can perform calculations in steps 2 and 3. **b**, When there is NE heterogeneity is low in the user data, box-top colors of steps 2 and 3 will remain yellow and the users will be alerted that "Low correlation observed between NE score and gene expression data. NE signature may not be reliable!".

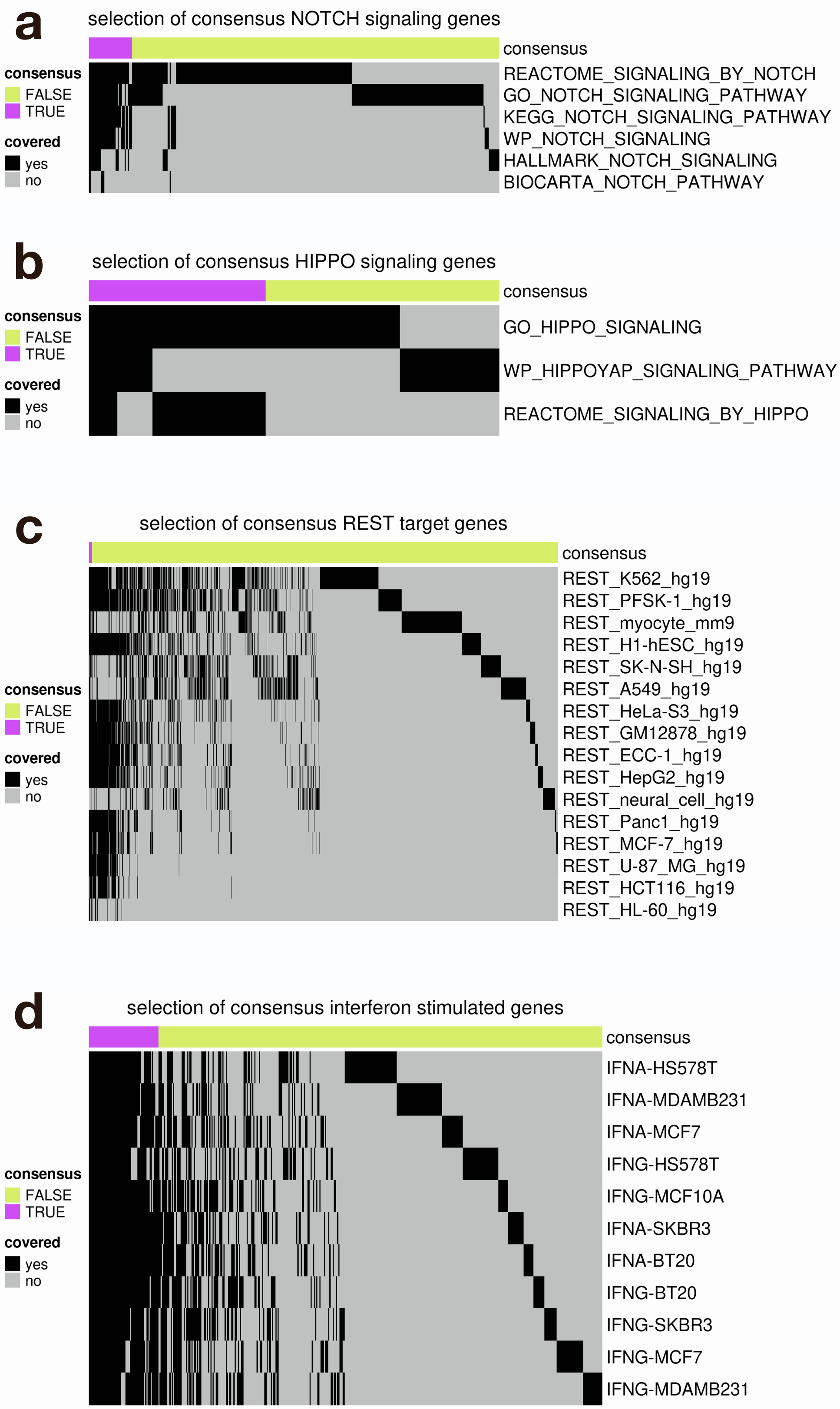

Figure S5. Deriving consensus gene sets, related to Figures 2-6.
Notch (**a**) and Hippo (**b**) pathway consensus genesets were determined from multiple related genesets in the canonical pathway and gene ontology library from MSigDB. A consensus gene for these two pathways was defined as a gene that is a member of at least half of the related genesets. REST targets (**c**) and interferon-stimulated genes (**d**) were determined from related genesets from Encode or LINCS. A consensus gene for these sets was defined as a gene that is a member of at least 4/5 of the related genesets.

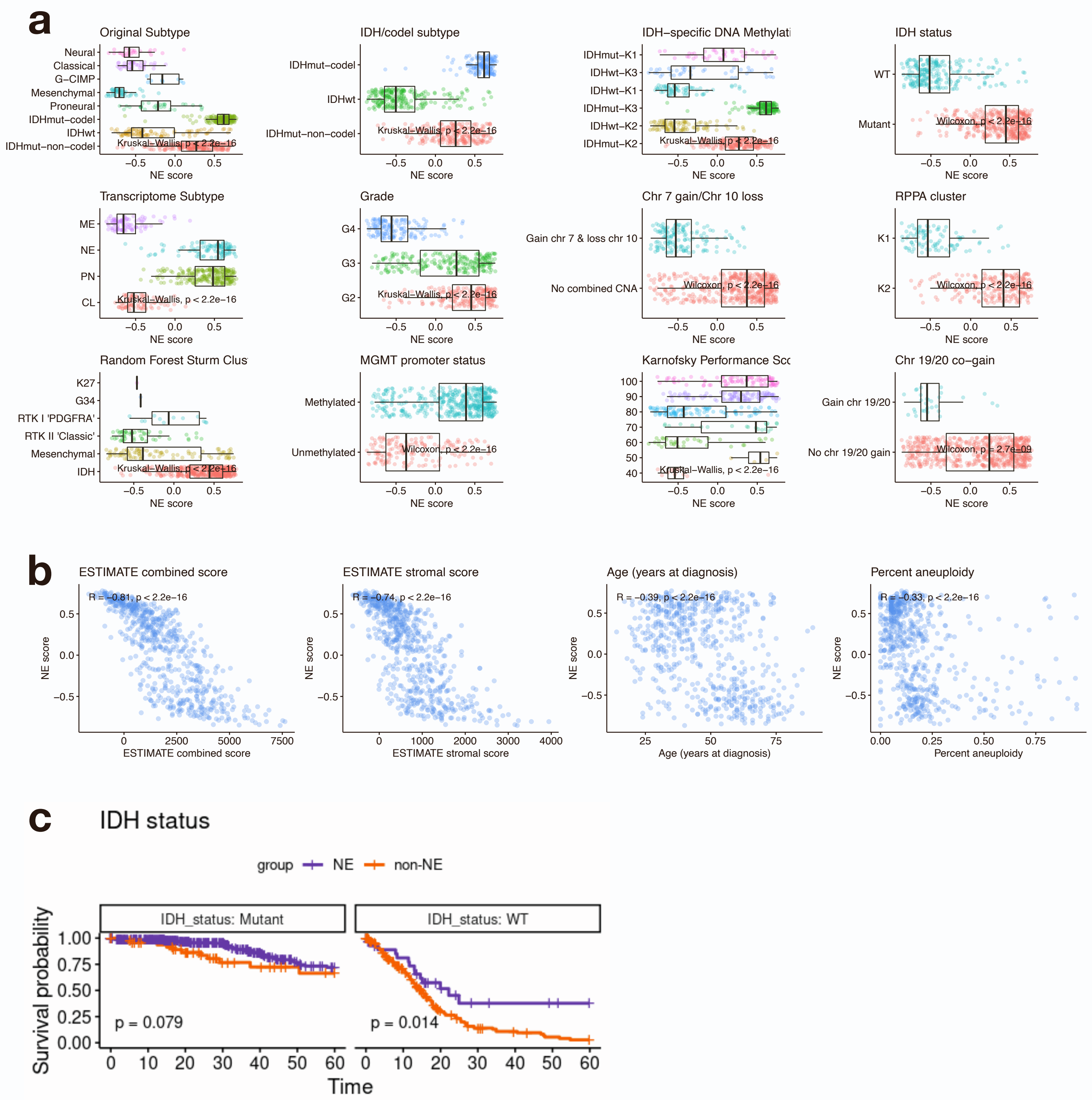

Figure S6. Significant associations between NE scores and orthogonal features in diffuse glioma, related to Figure 2
a, Association between NE scores and selected categorical features. **b**, Association between NE scores and selected continuous features. **c**, Low NE scores are associated with worse survival in IDH WT samples.

a

	HR	pval
'Telomere length estimate in tumor (Kb)'	0.6	0.019
	HR	pval
'Telomere length estimate in tumor (Kb)'	0.6	0.015
'NE score'	0.1	8.6e−11

b

	HR	pval
'TERT expression (log2)'	1.7	2.8e−16
	HR	pval
'TERT expression (log2)'	1.4	1.7e−07
'NE score'	0.1	1.9e−36

c

	HR	pval
'TERT expression status'Not expressed	0.4	4.8e−12
	HR	pval
'TERT expression status'Not expressed	0.4	1.8e−07
'NE score'	0.1	3e−39

d

	HR	pval
'TERT promoter status'WT	0.4	2.1e−05
	HR	pval
'TERT promoter status'WT	0.4	4.8e−05
'NE score'	0.2	1.3e−13

e

	HR	pval
'1p/19q codeletion'non−codel	6.3	1.6e−10
	HR	pval
'1p/19q codeletion'non−codel	1.4	0.29
'NE score'	0.1	1.5e−32

f

	HR	pval
'Chr 19/20 co−gain'No chr 19/20 gain	0.3	1.5e−07
	HR	pval
'Chr 19/20 co−gain'No chr 19/20 gain	0.9	0.75
'NE score'	0.1	4.4e−43

g

	HR	pval
'Chr 7 gain/Chr 10 loss'No combined CNA	0.1	1.8e−45
	HR	pval
'Chr 7 gain/Chr 10 loss'No combined CNA	0.4	1.2e−06
'NE score'	0.2	1.4e−16

h

	HR	pval
'MGMT promoter status'Unmethylated	4.1	6.4e−21
	HR	pval
'MGMT promoter status'Unmethylated	1.9	8.9e−05
'NE score'	0.1	1e−30

i

	HR	pval
'ABSOLUTE ploidy'	0.7	3.2e−05
	HR	pval
'ABSOLUTE ploidy'	0.8	0.009
'NE score'	0.1	3.9e−36

j

	HR	pval
'Percent aneuploidy'	1.4	1.3e−13
	HR	pval
'Percent aneuploidy'	1.1	0.023
'NE score'	0.1	2.8e−37

k

	HR	pval
'ATRX status'WT	3.3	1.2e−09
	HR	pval
'ATRX status'WT	2.6	1.7e−06
'NE score'	0.1	2.3e−39

l

	HR	pval
'BRAF V600E status'WT	0.2	0.035
	HR	pval
'BRAF V600E status'WT	0.5	0.4
'NE score'	0.1	3.5e−44

m

	HR	pval
'IDH status'WT	13.3	6.5e−54
	HR	pval
'IDH status'WT	7.5	8.8e−19
'NE score'	0.5	0.00021

n

	HR	pval
'IDH/codel subtype'IDHmut−non−codel	1.7	0.1
'IDH/codel subtype'IDHwt	19.7	2e−24
	HR	pval
'IDH/codel subtype'IDHmut−non−codel	1.3	0.49
'IDH/codel subtype'IDHwt	9.5	1.1e−09
'NE score'	0.5	0.00082

o

	HR	pval
'ESTIMATE stromal score'	1.9	1.4e−24
	HR	pval
'ESTIMATE stromal score'	0.9	0.14
'NE score'	0.1	5.3e−32

p

	HR	pval
'ESTIMATE immune score'	2	1.6e−25
	HR	pval
'ESTIMATE immune score'	0.8	0.069
'NE score'	0.1	1.9e−30

q

	HR	pval
'ESTIMATE combined score'	2	7.1e−27
	HR	pval
'ESTIMATE combined score'	0.8	0.073
'NE score'	0.1	4.3e−30

r

	HR	pval
StudyGlioblastoma multiforme	9.3	4.2e−54
	HR	pval
StudyGlioblastoma multiforme	3.3	8.4e−10
'NE score'	0.2	1.4e−12

s

	HR	pval
'Karnofsky Performance Score'	0.5	1.5e−21
	HR	pval
'Karnofsky Performance Score'	0.6	7e−08
'NE score'	0.2	3.1e−17

t

	HR	pval
'Age (years at diagnosis)'	2.8	1.9e−37
	HR	pval
'Age (years at diagnosis)'	2.1	3.3e−17
'NE score'	0.2	2.7e−23

u

	HR	pval
GradeG3	3.8	7.2e−07
GradeG4	23.2	5.3e−34
	HR	pval
GradeG3	3.1	2.5e−05
GradeG4	8.9	1.8e−12
'NE score'	0.3	1.6e−07

v

	HR	pval
Histologyglioblastoma	6.8	8.6e−25
Histologyoligoastrocytoma	0.6	0.11
Histologyoligodendroglioma	0.5	0.0031
	HR	pval
Histologyglioblastoma	3.5	5.3e−09
Histologyoligoastrocytoma	0.9	0.69
Histologyoligodendroglioma	0.8	0.47
'NE score'	0.3	1.6e−07

w

	HR	pval
'Pan−Glioma RNA Expression Cluster'LGr2	0.4	0.0066
'Pan−Glioma RNA Expression Cluster'LGr3	0.5	0.0023
'Pan−Glioma RNA Expression Cluster'LGr4	5.3	2.4e−17
	HR	pval
'Pan−Glioma RNA Expression Cluster'LGr2	0.5	0.046
'Pan−Glioma RNA Expression Cluster'LGr3	0.3	3.3e−07
'Pan−Glioma RNA Expression Cluster'LGr4	1	0.91
'NE score'	0.2	6.6e−11

y

	HR	pval
'IDH−specific RNA Expression Cluster'IDHmut−R2	0.5	0.14
'IDH−specific RNA Expression Cluster'IDHmut−R3	1.3	0.5
'IDH−specific RNA Expression Cluster'IDHwt−R1	10.8	7.2e−12
'IDH−specific RNA Expression Cluster'IDHwt−R2	15.5	1.9e−16
'IDH−specific RNA Expression Cluster'IDHwt−R3	14	3.8e−15
'IDH−specific RNA Expression Cluster'IDHwt−R4	10.1	4.6e−10
	HR	pval
'IDH−specific RNA Expression Cluster'IDHmut−R2	0.5	0.14
'IDH−specific RNA Expression Cluster'IDHmut−R3	0.7	0.41
'IDH−specific RNA Expression Cluster'IDHwt−R1	3	0.017
'IDH−specific RNA Expression Cluster'IDHwt−R2	4.1	0.0023
'IDH−specific RNA Expression Cluster'IDHwt−R3	6.1	5.1e−06
'IDH−specific RNA Expression Cluster'IDHwt−R4	5	0.00011
'NE score'	0.3	2e−05

aa

	HR	pval
'RPPA cluster'K2	0.1	7.9e−23
	HR	pval
'RPPA cluster'K2	0.3	1e−04
'NE score'	0.3	4.3e−05

x

	HR	pval
'Pan−Glioma DNA Methylation Cluster'LGm2	0.2	1.2e−05
'Pan−Glioma DNA Methylation Cluster'LGm3	0.3	0.0025
'Pan−Glioma DNA Methylation Cluster'LGm4	5.3	1.7e−08
'Pan−Glioma DNA Methylation Cluster'LGm5	6.8	2.3e−11
'Pan−Glioma DNA Methylation Cluster'LGm6	1.8	0.1
	HR	pval
'Pan−Glioma DNA Methylation Cluster'LGm2	0.3	7.6e−05
'Pan−Glioma DNA Methylation Cluster'LGm3	0.4	0.035
'Pan−Glioma DNA Methylation Cluster'LGm4	3.9	2.2e−05
'Pan−Glioma DNA Methylation Cluster'LGm5	5.1	2.4e−07
'Pan−Glioma DNA Methylation Cluster'LGm6	1.7	0.13
'NE score'	0.6	0.04

ab

	HR	pval
'Transcriptome Subtype'ME	1	1
'Transcriptome Subtype'NE	0.1	3.1e−14
'Transcriptome Subtype'PN	0.1	4.1e−23
	HR	pval
'Transcriptome Subtype'ME	0.8	0.27
'Transcriptome Subtype'NE	0.7	0.34
'Transcriptome Subtype'PN	0.6	0.13
'NE score'	0.2	1.2e−08

z

	HR	pval
'IDH−specific DNA Methylation Cluster'IDHmut−K2	0.2	1.3e−06
'IDH−specific DNA Methylation Cluster'IDHmut−K3	0.2	3.4e−06
'IDH−specific DNA Methylation Cluster'IDHwt−K1	4	1.7e−06
'IDH−specific DNA Methylation Cluster'IDHwt−K2	5.1	1.3e−08
'IDH−specific DNA Methylation Cluster'IDHwt−K3	1.4	0.37
	HR	pval
'IDH−specific DNA Methylation Cluster'IDHmut−K2	0.2	1.9e−06
'IDH−specific DNA Methylation Cluster'IDHmut−K3	0.2	9.5e−05
'IDH−specific DNA Methylation Cluster'IDHwt−K1	2.9	0.0013
'IDH−specific DNA Methylation Cluster'IDHwt−K2	3.7	6.2e−05
'IDH−specific DNA Methylation Cluster'IDHwt−K3	1.3	0.53
'NE score'	0.6	0.06

ac

	HR	pval
'Original Subtype'G−CIMP	0.3	0.035
'Original Subtype'IDHmut−codel	0	4.1e−21
'Original Subtype'IDHmut−non−codel	0.1	8.3e−26
'Original Subtype'IDHwt	0.5	0.0011
'Original Subtype'Mesenchymal	1.1	0.61
'Original Subtype'Neural	1.1	0.76
'Original Subtype'Proneural	1.2	0.53
	HR	pval
'Original Subtype'G−CIMP	0.3	0.084
'Original Subtype'IDHmut−codel	0.1	3.2e−09
'Original Subtype'IDHmut−non−codel	0.1	2e−13
'Original Subtype'IDHwt	0.5	0.013
'Original Subtype'Mesenchymal	1	0.86
'Original Subtype'Neural	1.1	0.73
'Original Subtype'Proneural	1.3	0.31
'NE score'	0.6	0.061

ae

	HR	pval
'Supervised DNA Methylation Cluster'Codel	0	1.7e−23
'Supervised DNA Methylation Cluster'G−CIMP−high	0.1	2.4e−31
'Supervised DNA Methylation Cluster'G−CIMP−low	0.5	0.064
'Supervised DNA Methylation Cluster'LGm6−GBM	1.8	0.12
Supervised DNA Methylation Cluster'Mesenchymal−like	1.3	0.18
'Supervised DNA Methylation Cluster'PA−like	0.1	2.4e−05
	HR	pval
'Supervised DNA Methylation Cluster'Codel	0	7.6e−13
'Supervised DNA Methylation Cluster'G−CIMP−high	0.1	2.8e−19
'Supervised DNA Methylation Cluster'G−CIMP−low	0.5	0.087
'Supervised DNA Methylation Cluster'LGm6−GBM	1.8	0.13
Supervised DNA Methylation Cluster'Mesenchymal−like	1.3	0.18
'Supervised DNA Methylation Cluster'PA−like	0.1	8.1e−05
'NE score'	1	0.91

ad

	HR	pval
'Random Forest Sturm Cluster'IDH	0	0.00042
'Random Forest Sturm Cluster'K27	0.6	0.72
'Random Forest Sturm Cluster'Mesenchymal	0.2	0.092
'Random Forest Sturm Cluster'RTK I 'PDGFRA'	0.2	0.18
'Random Forest Sturm Cluster'RTK II 'Classic'	0.4	0.43
	HR	pval
'Random Forest Sturm Cluster'IDH	0.1	0.0053
'Random Forest Sturm Cluster'K27	0.6	0.69
'Random Forest Sturm Cluster'Mesenchymal	0.2	0.17
'Random Forest Sturm Cluster'RTK I 'PDGFRA'	0.4	0.39
'Random Forest Sturm Cluster'RTK II 'Classic'	0.4	0.38
'NE score'	0.3	1e−05

Figure S7 CoxPH model including NE score as a covariate, related to Figure 2.
Hazard ratio (HR) and p-values from models with or without NE scores are compared for each prognostic molecular or pathological feature. Continuous features were z-transformed.

a

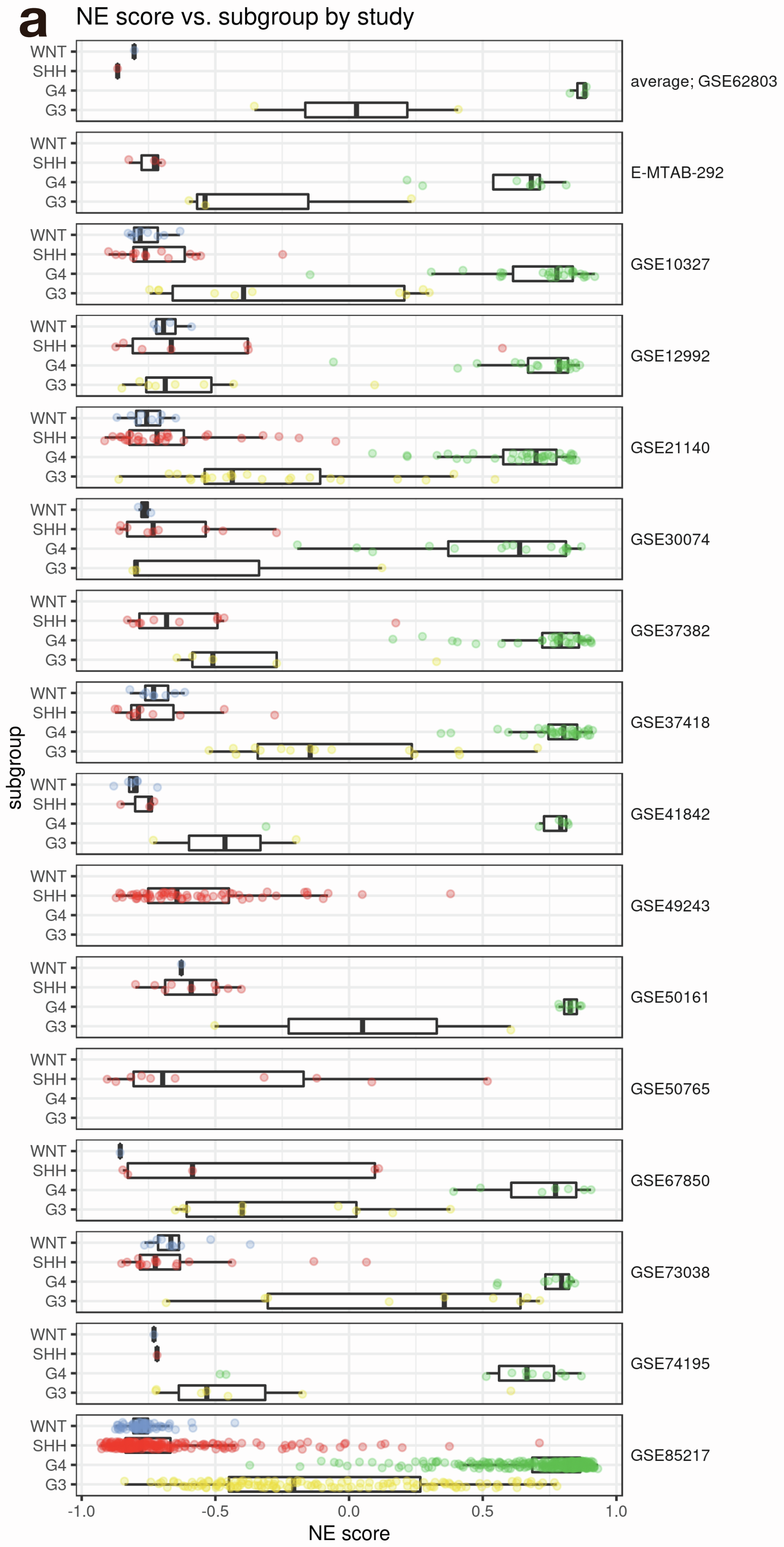

b

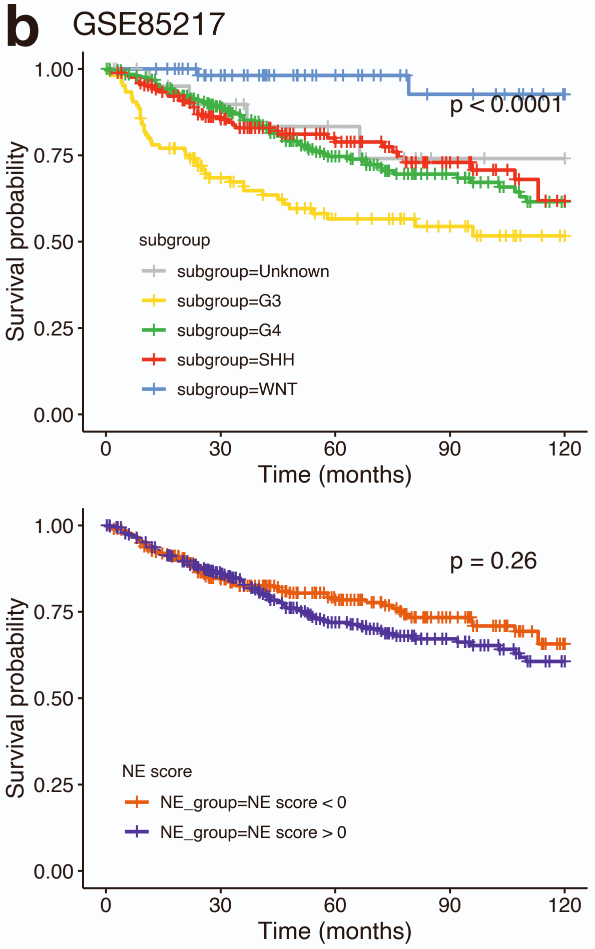

c CoxPH model for NE score and subgroup with interaction

	HR	pval
'NE score'	2.8	0.33
subgroupG3	3	0.086
subgroupG4	4.3	0.038
subgroupSHH	1.3	0.72
subgroupWNT	1.5	0.95
'NE score':subgroupG3	0.3	0.25
'NE score':subgroupG4	0.1	0.033
'NE score':subgroupSHH	0.3	0.34
'NE score':subgroupWNT	4.9	0.84

d Multivariate CoxPH model for G4 subgroup

	HR	pval
'NE score'	0.2	0.0022
'Metastatic stage'M+	1.2	0.45
GenderMale	1	0.96
Age	1	0.89

Figure S8. Detailed association analyses of NE scores and features of MB, related to Figure 3.
a, Relationship between NE scores and molecular subtypes in 16 different MB studies. **b**, Kaplan-Meier survival analysis of molecular subtypes and NE subgroups in MB patients from GSE85217. **c**, Results of a multivariate CoxPH regression model that included molecular subtype, NE scores, and their interaction term. Interaction between NE score and subgroup G4 is statistically significant. **d**, hazard ratio (HR) and p-value from multivariate CoxPH regression that included NE score, metastatic stage, gender, and age as covariates.

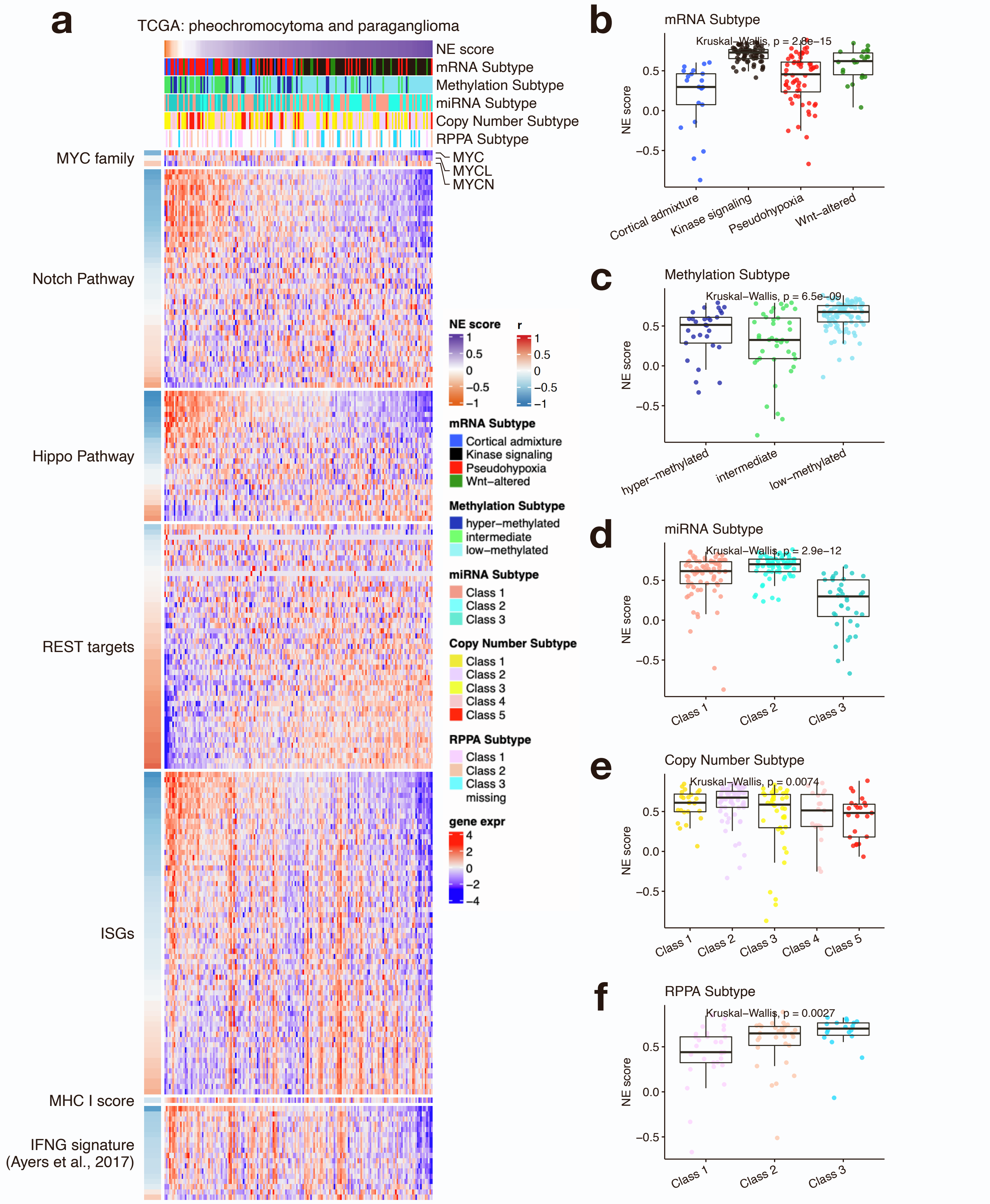

Figure S9. Aligning NE scores to various features of pheochromocytoma and paraganglioma (PCPG), related to Figure 1. **a**, Heatmap aligning NE scores to molecular subtypes and selected gene expression in PCPG. **b-f**, Comparison of NE scores across different mRNA clusters (**b**), methylation clusters (**c**), miRNA clusters (**d**), copy number clusters (**e**), and RPPA subtypes (**f**).

a

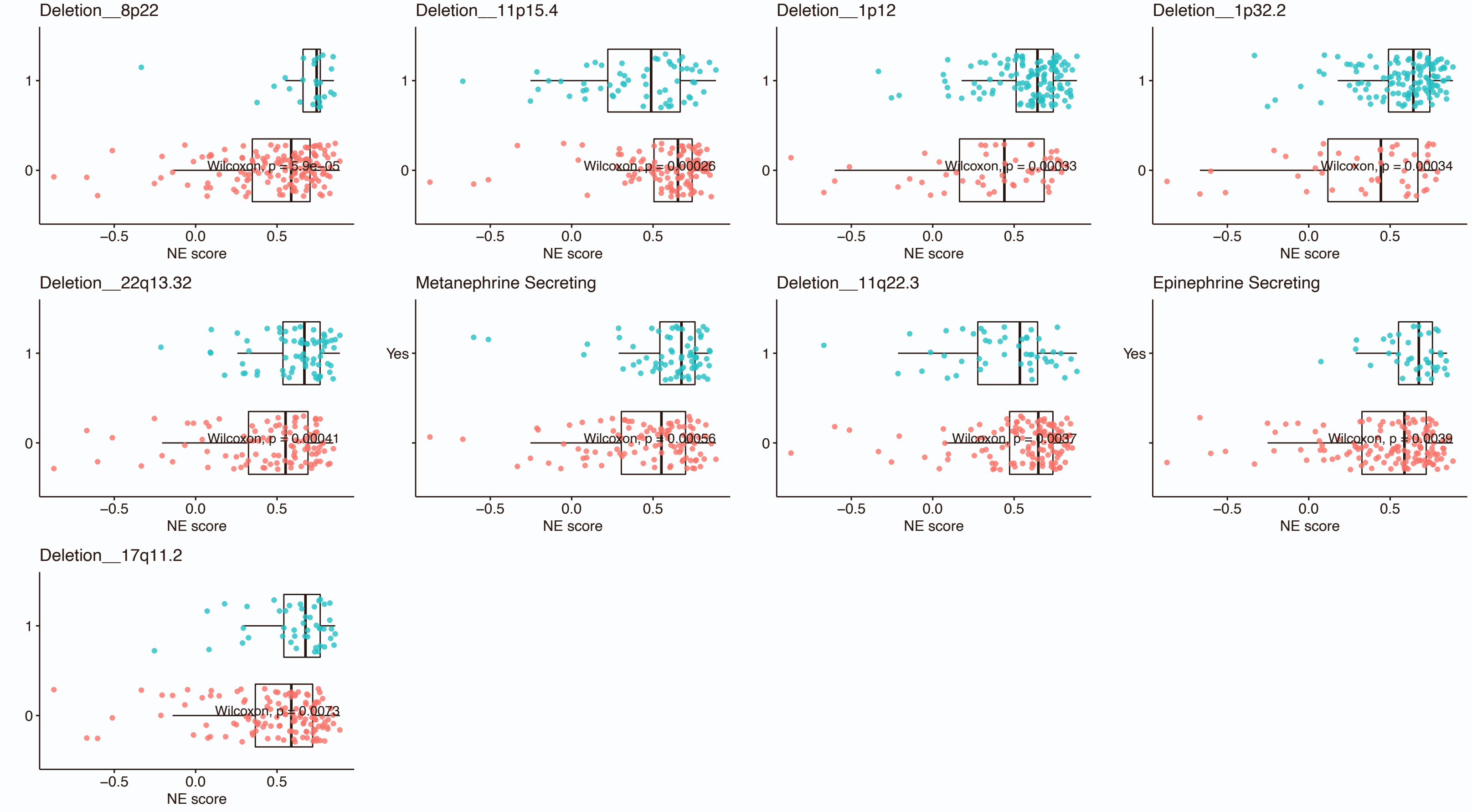

b

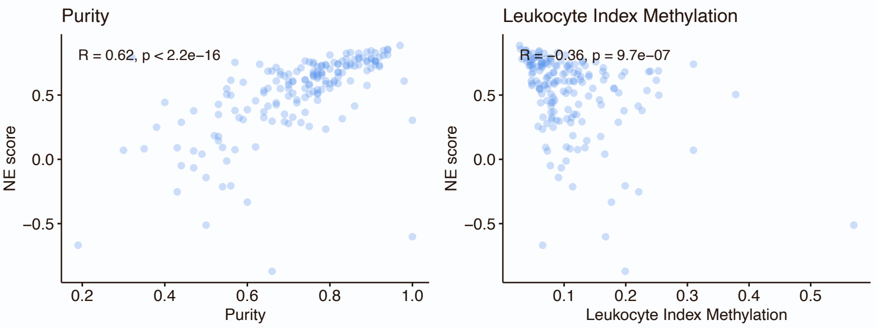

Figure S10. Significant associations between NE scores and orthogonal features in PCPG, related to Figure 1.
a, Association between NE scores and selected copy number alteration events and biochemical properties of PCPG. **b**, Association between NE scores and selected continuous features.

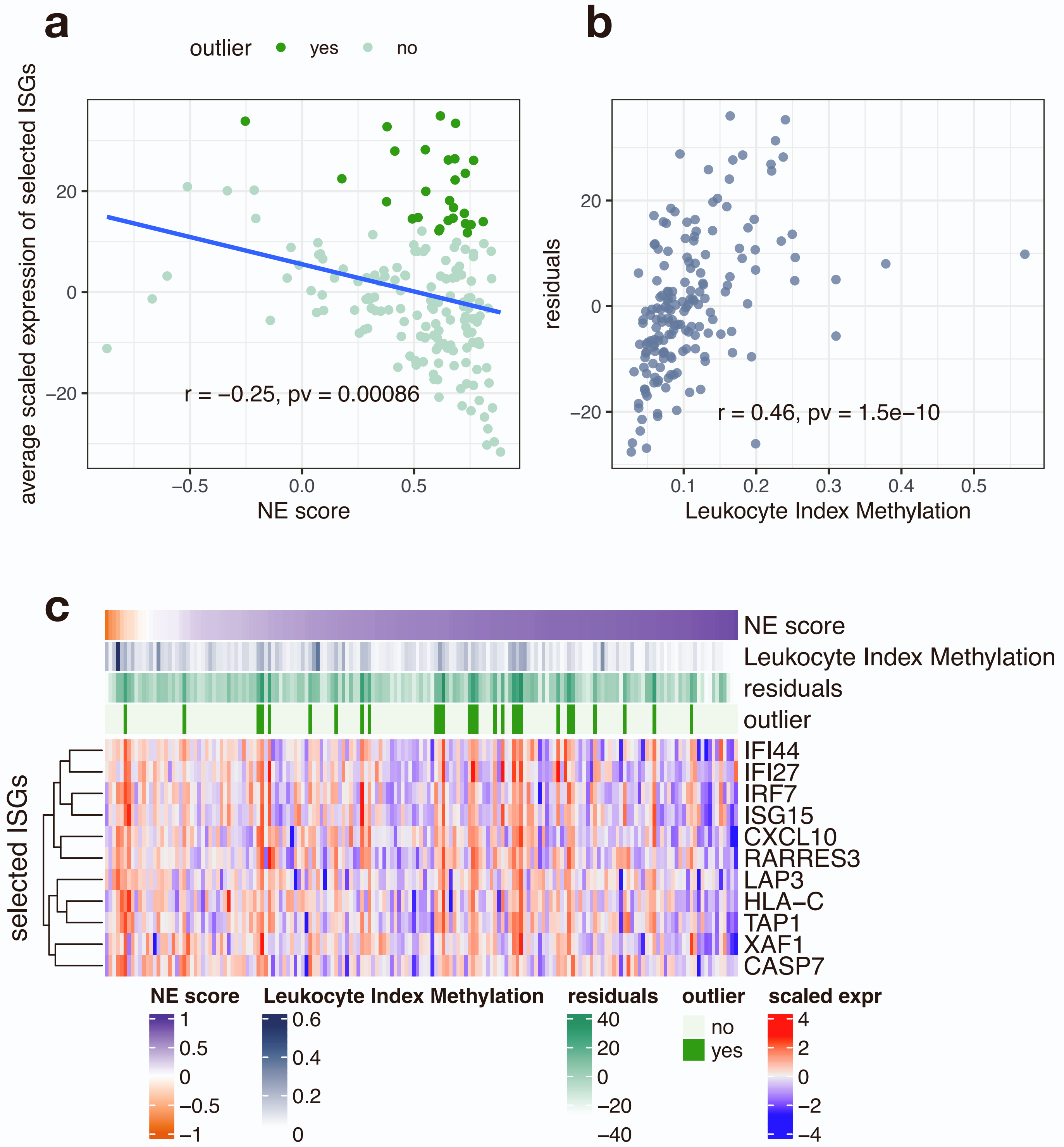

Figure S11. Leukocyte expression of ISGs confound ISG expression correlation with NE scores, related to Figure 1. **a**, Outlier identification from regressing average ISG expression on NE scores in PCPG samples. Model-based clustering was used to identify samples with outlier residuals. **b**, Positive correlation between residuals from linear model in (**a**) and leukocyte index methylation (tumor-infiltrating leukocyte estimate). **c**, Alignment of NE score, leukocyte index methylation, residuals and outliers from **a**, and expression of selected ISGs.

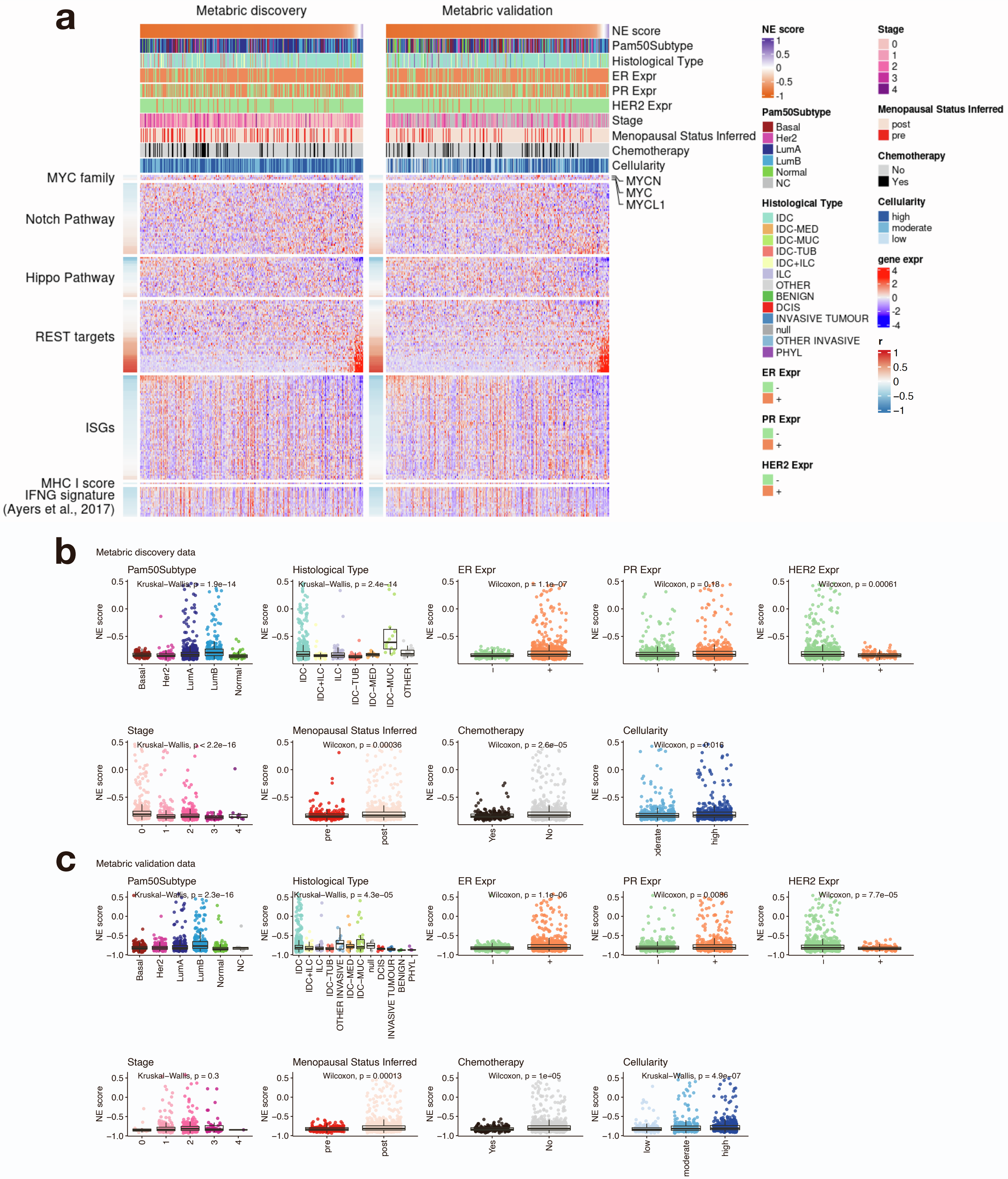

Figure S12. Aligning NE scores to various features of breast cancer from the Metabric discovery and validation datasets, related to Figure 5.
a, Heatmap aligning NE scores to molecular subtypes, pathological subtypes, and selected gene expression in discovery and validation datasets from Metabric. **b-c**, Comparison of NE scores across different features in discovery (**b**) and validation (**c**) datasets.

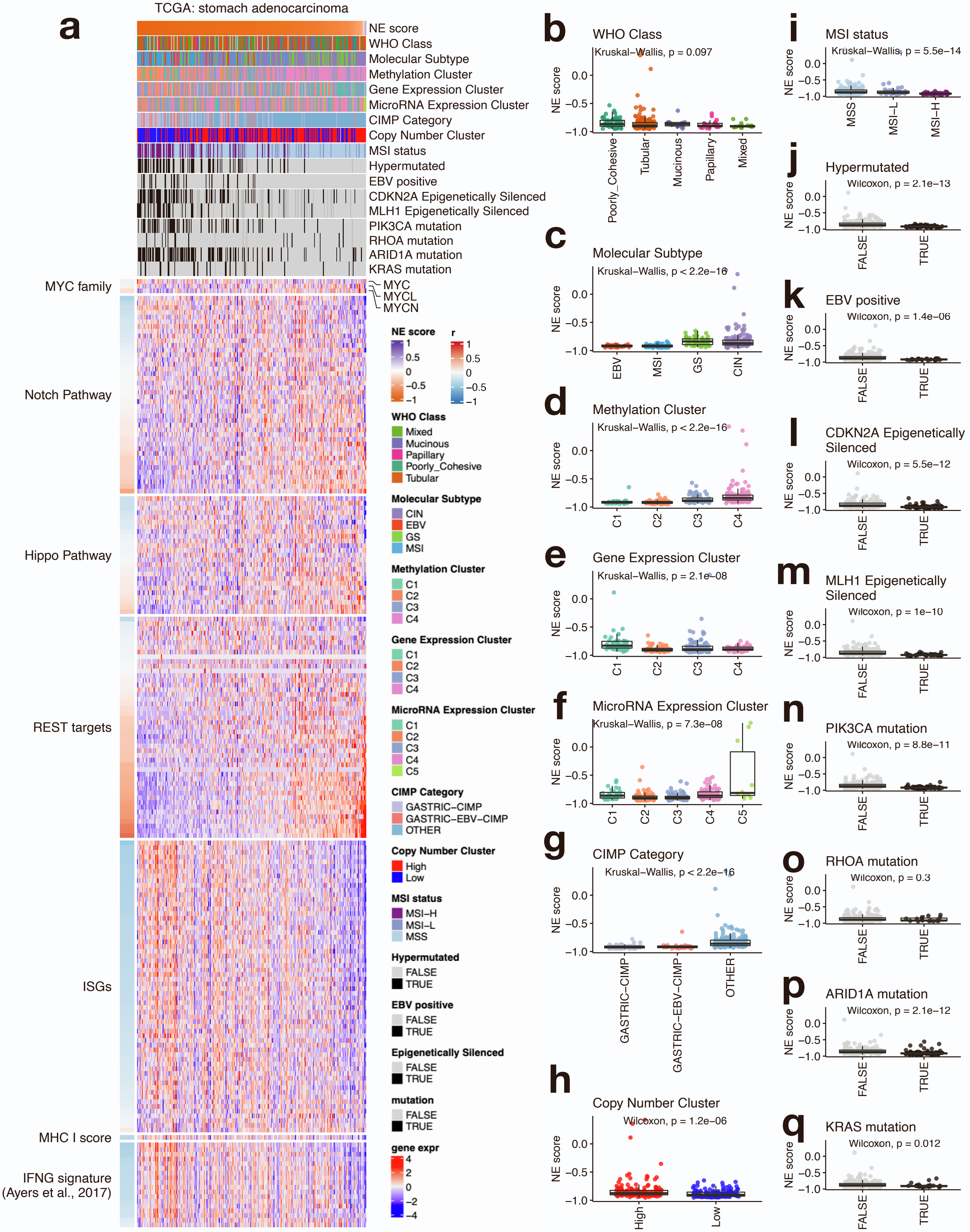

Figure S13. Aligning NE scores to various features of stomach adenocarcinoma (STAD), related to Figure 1.
a, Heatmap aligning NE scores to histological, molecular subtypes, selected genetic alterations and selected gene expression in STAD. **b-q**, Comparison of NE scores across different histological subtypes (**b**), molecular subtypes (**c**), methylation (**d**), RNA (**e**), miRNA (**f**) clusters, CIMP categories (**g**), copy number classes (**h**), MSI (**i**), hypermutation (**j**), EBV (**k**) status, epigenetic silencing status of *CDNK2A* (**l**) and *MLH1* (**m**), and mutation status of *PIK3CA* (**n**), *RHOA* (**o**), *ARID1A* (**p**), and *KRAS* (**q**).

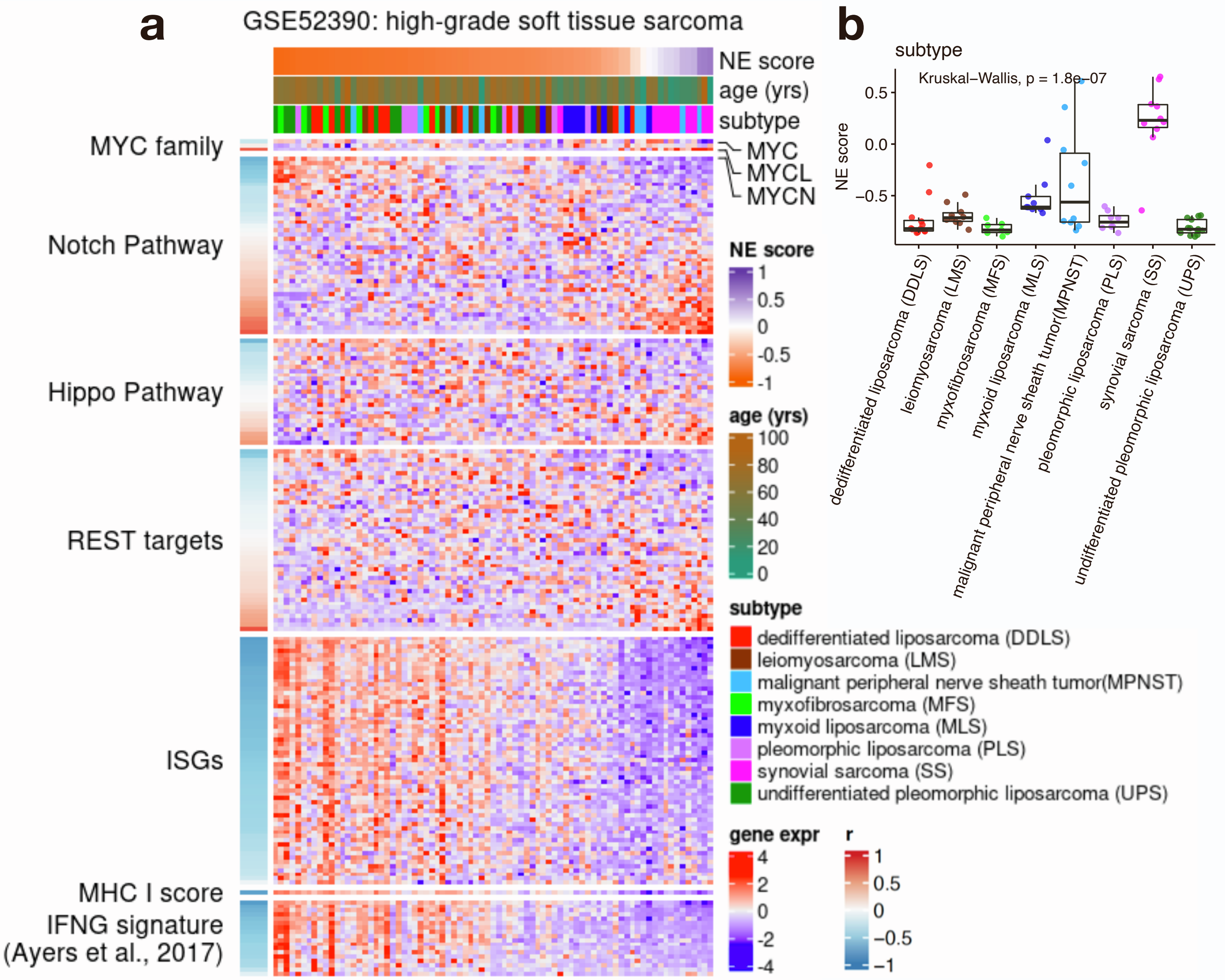

Figure S14. Aligning NE scores to various features of high-grade soft tissue sarcoma, related to Figure 1.
a, Heatmap aligning NE scores to sarcoma subtypes and age. **b**, Detailed comparison of NE scores in different sarcoma subtypes.
